# The genome and developmental transcriptome of the strongylid nematode *Haemonchus contortus*

**DOI:** 10.1186/gb-2013-14-8-r89

**Published:** 2013-08-28

**Authors:** Erich M Schwarz, Pasi K Korhonen, Bronwyn E Campbell, Neil D Young, Aaron R Jex, Abdul Jabbar, Ross S Hall, Alinda Mondal, Adina C Howe, Jason Pell, Andreas Hofmann, Peter R Boag, Xing-Quan Zhu, T Ryan Gregory, Alex Loukas, Brian A Williams, Igor Antoshechkin, C Titus Brown, Paul W Sternberg, Robin B Gasser

**Affiliations:** 1 Howard Hughes Medical Institute and Division of Biology, California Institute of Technology, Pasadena, California 91125, USA; 2 Faculty of Veterinary Science, The University of Melbourne, Corner of Flemington Road and Park Drive, Parkville, Victoria 3010, Australia; 3 Current address: Department of Molecular Biology and Genetics, Cornell University, Ithaca, New York, 14853-2703, USA; 4 Department of Microbiology and Molecular Genetics, Michigan State University, East Lansing, Michigan, 48824, USA; 5 Department of Computer Science and Engineering, Michigan State University, East Lansing, Michigan, 48824, USA; 6 Eskitis Institute for Cell and Molecular Therapies, Griffith University, N75 Don Young Road, Brisbane Innovation Park, Nathan, Queensland 4111, Australia; 7 Faculty of Medicine, Nursing and Health Sciences, Monash University, Wellington Road, Clayton, Victoria 3800, Australia; 8 State Key Laboratory of Veterinary Etiological Biology, Lanzhou Veterinary Research Institute, Chinese Academy of Agricultural Sciences, 1 Xujiaping, Yanchangbu, Lanzhou, Gansu Province 730046, PR China; 9 Department of Integrative Biology, University of Guelph, Ontario, Canada N1G 2W1; 10 Center for Biodiscovery and Molecular Development of Therapeutics, Queensland Tropical Health Alliance, James Cook University, Cairns, Queensland 4870, Australia

## Abstract

**Background:**

The barber's pole worm, *Haemonchus contortus*, is one of the most economically important parasites of small ruminants worldwide. Although this parasite can be controlled using anthelmintic drugs, resistance against most drugs in common use has become a widespread problem. We provide a draft of the genome and the transcriptomes of all key developmental stages of *H. contortus *to support biological and biotechnological research areas of this and related parasites.

**Results:**

The draft genome of *H. contortus *is 320 Mb in size and encodes 23,610 protein-coding genes. On a fundamental level, we elucidate transcriptional alterations taking place throughout the life cycle, characterize the parasite's gene silencing machinery, and explore molecules involved in development, reproduction, host-parasite interactions, immunity, and disease. The secretome of *H. contortus *is particularly rich in peptidases linked to blood-feeding activity and interactions with host tissues, and a diverse array of molecules is involved in complex immune responses. On an applied level, we predict drug targets and identify vaccine molecules.

**Conclusions:**

The draft genome and developmental transcriptome of *H. contortus *provide a major resource to the scientific community for a wide range of genomic, genetic, proteomic, metabolomic, evolutionary, biological, ecological, and epidemiological investigations, and a solid foundation for biotechnological outcomes, including new anthelmintics, vaccines and diagnostic tests. This first draft genome of any strongylid nematode paves the way for a rapid acceleration in our understanding of a wide range of socioeconomically important parasites of one of the largest nematode orders.

## Background

The strongylid nematode *Haemonchus contortus *(barber's pole worm) is one of the most important parasites of livestock, and represents a large order of nematodes (Strongylida) that infect both animals and humans worldwide [1-3]. *H. contortus *infects hundreds of millions of sheep and goats globally, and causes deaths and production losses estimated at tens of billions of dollars per annum. This nematode feeds on blood from capillaries in the stomach mucosa, and causes hemorrhagic gastritis, anemia, edema and associated complications, often leading to death of severely affected animals [2]. *H. contortus *is transmitted orally from contaminated pasture to the host through a complex 3-week life cycle [4]: the eggs are excreted in the host feces, the first-stage larva (L1) develops inside the egg, then hatches (within about 1 day) and molts through to the second-stage (L2) and third-stage (L3) larval stages within approximately 1 week; the infective L3s are then ingested by the host, exsheath, and, after a histotropic phase, develop through the fourth-stage larvae (L4s) to dioecious adults; these two last stages both feed on blood.

Only four main drug classes have been available for the treatment of strongylid infections, and resistance against these classes is spreading worldwide [5]. It is thus highly desirable to search for new drug targets encoded in the *H. contortus *genome. Although vaccines using some native parasite antigens (called H11 or H-gal-GP) can partially prevent haemonchosis in experimental sheep, homologous recombinant molecules have failed to achieve protection [6]. Therefore, current treatment relies predominantly on the use of nematocidal drugs (anthelmintics). Because resistance against the main classes of drugs has become widespread [5], the ongoing design of new compounds, such as monepantel [7], cyclooctadepsipeptides [8], and derquantel-abamectin [9], is required. Discovering new drugs has been challenging, particularly because of the current limited understanding of the biology of *H. contortus *and how it interacts with its host [2]. Here, we describe a draft genome and developmentally staged transcriptome of *H. contortus *to substantially improve our understanding of this parasite at the molecular level. This genome provides a major resource to the scientific community for a wide range of genomic, genetic, evolutionary, biological, ecological, and epidemiological investigations, and a solid foundation for the development of new interventions (drugs, vaccines and diagnostic tests) against *H. contortus *and related strongylid nematodes.

## Results and discussion

### Sequencing and assembly

We sequenced the genome of *H. contortus *(McMaster strain, Australia) at 185-fold coverage (Table 1; see Additional file 1 Table S1), producing a final draft assembly of 320 Mb (scaffold N50: 56.3 kb; Table 1) with a mean GC content of 42.4%. We detected 91.5% of 248 core essential genes by CEGMA, suggesting that the assembly represents a substantial proportion of the entire genome. The estimated repeat content for this draft genome is 13.4%, equating to 42.8 Mb DNA. To overcome challenges in the assembly of the genome, we removed excessive repetitive and erroneous reads by *khmer *filtering [10] and normalization [11] to produce a representative assembly, an approach that should be useful for other complex genomes. This assembly contained 2.0% retrotransposons and 2.1% DNA transposons, which is similar to that reported for some other nematode genomes sequenced to date, including those of *Caenorhabditis elegans, Pristionchus pacificus*, and *Ascaris suum *[12-14]. We identified 40,046 retrotransposon sequences (see Additional file 1, Table S2) representing at least 9 families (4 long terminal repeats (LTRs), 3 long interspersed nuclear elements (LINEs), and 2 short interspersed nuclear elements (SINEs)). We also identified two families of DNA transposons (36,861 distinct sequences in total) and 235,635 unclassified repeat elements. The most abundantly transcribed repeat elements were DNA/TcMar and LINEs/retrotransposable elements (RTEs; see Additional file 1, Table S2). This richness of transposable element families is substantially higher than that predicted for other genomes of parasitic nematodes [13-16]. Overall, the present draft genome (320 Mb) is the largest of any animal parasitic nematode sequenced to date (for example, 273, 89, and 64 Mb for *A. suum, Brugia malayi*, and *Trichinella spiralis*, respectively)[14-16].

**Table 1:** Features of the *Haemonchus contortus *draft genome

Description	
Total number of base pairs within assembled scaffolds	319,640,208
Total number of scaffolds; contigs	14,419; 930,981
N50 length in bp; total number > 2 kb in length	56,328; 11,000
N90 length in bp; total number > N90 length	13,105; 6,085
GC content of the whole genome (%)	42.4
Repetitive sequences (%)	13.4
Proportion of genome that is coding (exonic; incl. introns) (%)	8.6; 43.3
Number of putative coding genes	23,610
Gene size (mean bp)	6,167
Average coding domain length (mean bp)	835
Average exon number per gene (mean)	7
Gene exon length (mean bp)	139
Gene intron length (mean bp)	832
GC content in coding regions (%)	45.4
Number of transfer RNAs	449

### *H. contortus *gene set

**Figure 1:**
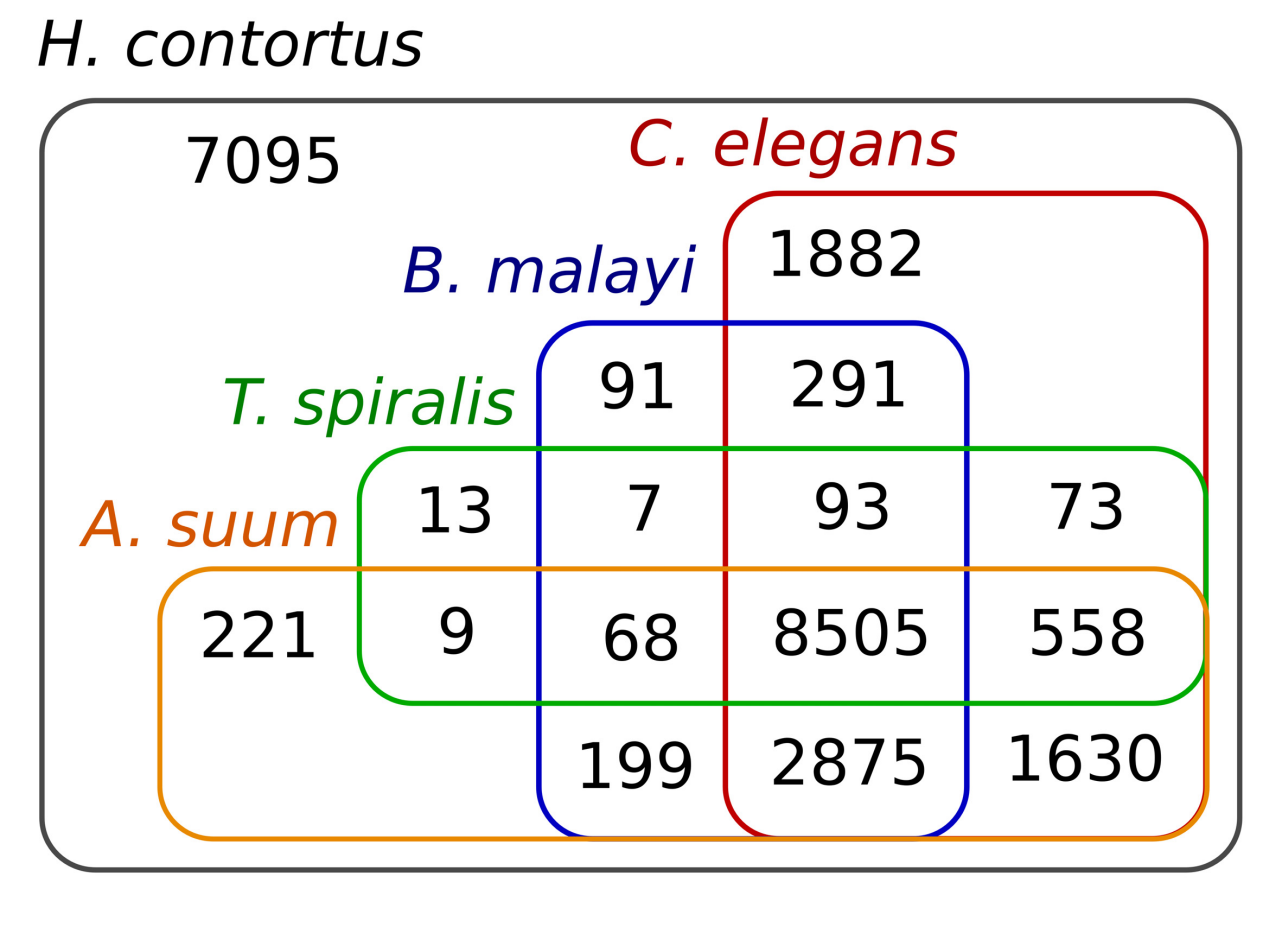
**Venn diagram **showing the numbers of homologs between *Haemonchus contortus *and four other nematode species (*Ascaris suum, Brugia malayi, Caenorhabditis elegans, *and *Trichinella spiralis*) after pairwise comparison.

Using transcriptomic data from egg, larval, and adult stages of *H. contortus *(Haecon-5 strain, Australia), *de novo *predictions and homology-based searching, we annotated 23,610 genes, all of which are supported by transcriptomic and protein data, with a mean total length of 6,167 bp, exon length of 139 bp, and estimated 7.2 exons per gene (Table 1). Mean gene and intron lengths (6,167 and 832 bp) for *H. contortus *were comparable with those of *A. suum *(6,536 and 1,081 bp), but greater than those for other nematodes, such as *C. elegans, B. malayi*, and *T. spiralis *(around 1,000 to 2,000 and around 100 to 400 bp). Most of the predicted *H. contortus *genes (Figure 1) were found to have homologs (BLASTp e-value cut-off 10^-5^) in other nematodes (16,545; 70.0%), including *C. elegans *(15,907; 67.4%), *A. suum *(14,065; 59.6%), *B. malayi *(12,129; 51.4%), and *T. spiralis *(9,326; 39.5%). In total, 8,505 genes were found to be orthologous among the five species, with 608 being shared with at least one other species of nematode but absent from *C. elegans *(Figure 1). Conversely, 7,095 genes (30.1%) were found to be unique to *H. contortus *relative to the other four species (Figure 1). Conspicuous were at least 325 genes that are exclusive to all four parasitic nematodes and that are included here for comparison (≤10^-5^). Of the entire *H. contortus *gene set, 5,213 genes had an ortholog (≤10^-5^) linked to one of 291 known biological pathways (Kyoto Encyclopedia of Genes and Genomes; KEGG; see Additional file 1, Table S3). Mapping to pathways in *C. elegans *suggested a near-complete complement of genes, also supporting the CEGMA results. By inference, essentially all of the *H. contortus *genes are represented in the present genomic assembly, and are supported by extensive transcriptomic and inferred protein data. Using data for *C. elegans *and data available in all accessible protein- and/or conserved protein domain-databases, we predicted functions (including enzymes, receptors, channels, and transporters) for 19,391 (77.92%) of the protein-coding genes of *H. contortus *(Table 2).

**Table 2:** Major protein groups representing the *Haemonchus contortus *gene set

Protein group^a^	Number predicted
Channels	2,454
Ligand-gated ion channels (LGICs)	297
G protein-coupled receptors (GPCRs)	540
GTPases	247
Major sperm proteins (MSPs)	42
Vitellogenins	3
Peptidases	429
Peptidase inhibitors	119
Kinases	845
Phosphatases	330
RNAi machinery	229
Secretome	1,457
SCP/TAPS	84
Structural proteins	943
Other proteins with known homologues and/or domains	11,710
Hypothetical proteins	5,378

^a^Some predicted proteins belonged to multiple categories.

We identified 429 peptidases representing five key classes (aspartic, cysteine, serine, and threonine peptidases and metallopeptidases), with the metallopeptidases (*n *= 141; 32.9%) and serine peptidases (107; 24.9%) predominating (see Additional file 1, Table S4). Notable were secreted peptidases, such as astacins (M12A), neprilysins (M13), selected serine peptidases (SC; S09), cathepsins (C01A), and calpain-2s (C19), which are abundantly represented and, based on information available for other nematode species [13-15,17], likely to have key roles in host invasion, locomotion, migration into stomach tissue (during the histotropic phase), degradation of blood and other proteins, immune evasion, and/or activation of inflammation. We also identified 845 kinases and 330 phosphatases in *H. contortus *(see Additional file 1, Tables S5 and S6). All major classes of kinases are represented, with tyrosine kinase (*n * = 92), casein kinase 1 (*n * = 90), CMGC (*n * = 67), and calcium/calmodulin-dependent protein kinase (*n *= 65) homologs being abundant (37.2%), and a similar number of unclassified kinases (37%). The phosphatome includes mainly protein tyrosine (*n *= 69), serine/threonine (*n *= 50), receptor type tyrosine (*n *= 32), histidine (*n *= 31), and dual-specificity (*n *= 25) phosphatases. Based on homology with *C. elegans *proteins, we predicted 247 GTPases, including 215 small GTPases representing the Rho (*n *= 50), Rab (*n *= 38), Ran (*n *= 57), Arf (*n *= 23), and Miro (*n *= 2) families, and a small number of large GTPases (such as dyamin, GBP, and mitofusin; *n *= 15; see Additional file 1, Table S7). Examples of small GTPase homologs are *arf-1.2, eef-2 *and *tba-2*, whose *C. elegans *orthologs are essential for embryonic, larval, and/or reproductive development. Therefore, some of these enzymes were proposed as targets for anti-parasite interventions [18,19]. Similarly, the large range of channel, pore, and transporter proteins that we identified here is of particular interest in this context, considering that many common anthelmintics bind representatives of some of these proteins as targets [7]. For *H. contortus*, we predicted 540 G protein-coupled receptors (GPCRs), most of which belonged to classes SR (*n *= 299) and A (147; see Additional file 1, Table S8). In addition, we identified 786 channel or pore proteins, including voltage-gated ion channels (VICs) and ligand-gated ion channels (LGICs; see Additional file 1, Table S9). Such channels are known targets for nematocidal drugs, such as macrocyclic lactones (for example, cydectin), levamisole and monepantel (an aminoacetonitrile derivative; AAD) [7]. Importantly, in the *H. contortus *gene set, we found a homolog *acr-23 *of the *C. elegans *monepantel receptor, supporting evidence that this drug kills *H. contortus in vivo *[7]. In addition, we detected an abundance of transporters, including 617 electrochemical potential-driven (almost all porters) and 526 primary active (mainly P-P-bond-hydrolysis-driven) transporters, and 308 transport-associated molecules (see Additional file 1, Table S9).

Excretory/secretory (ES) proteins are central to the parasite-host relationship [19]. We predicted the secretome of *H. contortus *to comprise 1,457 proteins with a diverse range of functions (see Additional file 1, Table S10). Most notable were 318 peptidases, including 98 metallopeptidases and 68 cysteine, 67 aspartic, 19 serine peptidases (predominantly clans MA, CA, AA, and SA, respectively) and 66 peptidase inhibitors (including fibronectin type III), 90 lectins (including C-type and concanavalin A-like), 65 sperm-coating protein/Tpx-1/Ag5/PR-1/Sc7 (SCP/TAPS) proteins, 38 transthyretin-like (TTL) proteins, and 27 kinases. Many secreted peptidases (comprising the 'degradome') and their respective inhibitors have known roles in the penetration of tissue barriers and feeding for a range of parasitic worms, including *H. contortus *[2,6,18]. Some of these ES proteins are involved in host interactions and/or inducing or modulating host immune responses against parasitic worms, which are often Th2-biased [19].

### Key transcriptional changes during developmental transitions in the life cycle

**Figure 2:**
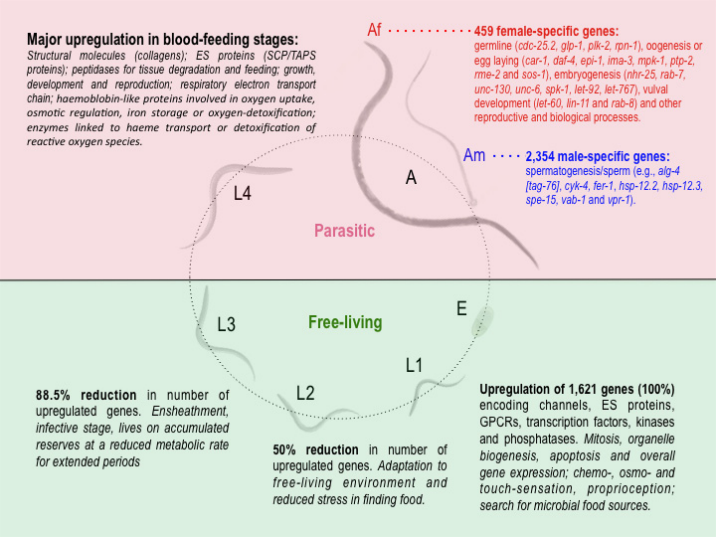
**Transcriptional changes in the life cycle of *Haemonchus contortus***. In a 3-week life cycle of the parasite, eggs (E) are excreted in host feces; the first-stage larva (L1) develops inside the egg to hatch and molt through to the second-stage (L2) and third-stage (L3) larval stages within a week. The infective L3s are then ingested by the small ruminant host, where they exsheath and, after a short tissue phase, develop through the fourth-stage larval (L4) stage to dioecious adults; both of these stages feed on host blood from capillaries in the internal wall of the stomach. Disease in the host relates to this blood-feeding activity. In this figure, changes in transcription in the transition from stage to stage are summarized and interpreted in the context of the biology of the parasite. Information is given on key genes differentially transcribed between adult female (Af) and male (Am) *H. contortus*, and involved in reproductive and other biological processes; gene codes follow those of *Caenorhabditis elegans *orthologs.

*H. contortus *development involves a number of tightly timed processes [4]. Embryogenesis generates the basic tissue types of the nematode, and each tissue type differentiates at a specific point in the developmental cycle. Post-embryonic structures required for parasitism and reproduction then differentiate in the larval stages L1 to L4. This includes the specialized development of the buccal capsule for blood feeding (from L4 onward), sexual differentiation at the L4 stage, and gametogenesis in the adult stage. Substantial growth occurs at the L2, L4, and adult stages. Development occurs in two different environments, on pasture for the free-living stages L1 to L3, and in the host for the dioecious L4 and adult stages (Figure 2). Each of these stages has different requirements, in terms of motility, sensory perception, metabolism, and the regulation of hormones of the endocrine system. L3, which is the infective stage, and thus represents the transitional stage from a free-living to parasitic organism, persists in the environment until it is ingested by the host, where it then receives a signal (mainly CO_2_) to commence its development as a parasite. The complexity of the *H. contortus *life cycle coincides with key developmental alterations in the nematode that probably require tightly controlled and rapidly regulated transcriptional changes.

We studied differential transcription from stage to stage, as the parasite developed from egg to adult (Figure 2). The transition from the undeveloped egg to L1 was associated with significant upregulation of transcription for 1,621 genes encoding a substantial number of channels (*n *= 641), including LGICs and ES proteins (*n *= 397), GPCRs (134), transcription factors (TFs; *n *= 43), kinases (*n *= 100), and phosphatases (*n *= 35; see Additional file 1, Table S11). Although this expansion is probably associated with mitosis, organelle biogenesis, apoptosis, and overall gene expression during the rapid growth and development of L1 [4], based on knowledge of *C. elegans *[20], the expansion of some key subsets of channels, pore and electrochemical potential-driven transporters, GPCRs (classes A and SR), and various kinases/phosphatases probably relate to chemosensation, mechanosensation, osmosensation, and/or proprioception of the free-living L1, as it rapidly adapts to its new and harsh external environment.

The activity of L1s of *H. contortus *and their search for microbial food sources might reflect the expansion of ES proteins and associated peptidases and their inhibitors (Figure 2). The switch to L2 sees an approximately 50% reduction in number of upregulated genes of the same groups, possibly reflecting the gradual adaptation to its free-living environment and a reduced level of stress in finding food. The transition to the L3 stage sees an 88.5% reduction in the number of differentially transcribed genes representing the same groups (Figure 2), an expected finding, given that this stage undergoes ensheathment, is no longer able to feed, and must live on accumulated reserves at a reduced metabolic rate in order to survive (as an 'arrested' but motile infective L3 stage) for extended periods in the external environment [2]. Once ingested by the host animal, the transition from the L3 to the parasitic L4 and adult stages sees a renewed, massive surge in the number of differentially transcribed genes of the same spectrum of molecules and of structural proteins, but, as expected, very limited differences between the L4 and adult stages, with the exception of some genes (for example, those encoding vitellogenin) that appear to relate specifically to reproduction (Figure 2; see Additional file 1, Table S11).

During the key transitions in the life cycle (from egg to L2, and then from L3 to L4) linked to substantial growth and development [2], a range of genes encoding collagens and cuticular proteins are upregulated per transition (Figure 2; see Additional file 1, Table S11). In the nematode cuticle, such molecules are crucial for the maintenance of nematode body shape, and also for protection against and contact with the external environment or host interface. We found prominent variation in transcription profiles among 28 individual collagen genes in the transition from the free-living to parasitic stages, consistent with previous immunoproteomic findings [21].

More than 120 peptidase genes were significantly upregulated in blood-feeding stages (Figure 2; see Additional file 1, Table S11). Conspicuous among them were genes encoding secreted peptidases of various clans, including MA (metallopeptidases; M12A, M01, M13, M12A, M10A), AA (aspartic peptidases; all A01A) and CA (cysteine peptidases; mostly CA01A; see Additional file 1, Table S11), which have known roles in the degradation of tissues during the parasite's histotropic phase and digestion of blood components following establishment and buccal-capsule attachment to the abomasal wall, and might be crucial for growth, development, and survival of *H. contortus *in the host animal [2]. These findings support previous evidence showing that, for example, cysteine peptidases play a crucial role in the catabolism of globin by the cleavage of hemoglobin in blood-feeding nematodes [22-25]. Concomitantly, in the blood-feeding stages, we observed upregulated transcription of genes encoding succinate dehydrogenase subunit B and glutamate dehydrogenase genes via the respiratory electron transport chain [26] (proposed to maintain the redox balance in response to the accumulation of the end products from anaerobic metabolism [27]), and hemoglobin-like proteins [28] (probably involved in oxygen uptake, osmotic regulation, iron storage and/or oxygen-detoxification [29]). We also found increased transcription of genes encoding enzymes, including glutathione S-transferase, cytoplasmic Cu/Zn superoxide dismutase, catalase, glutathione peroxidase and/or peroxiredoxin, which are likely to have roles in heme transport or detoxification of reactive oxygen species from endogenous metabolic activities from the host during *H. contortus *infection; this is supported by findings from previous investigations [30-32] and recognized as characteristic of tissue-dwelling or blood-dwelling parasites [32].

The initiation of reproduction in adult *H. contortus *was marked by a developmentally regulated transcription of sex-enriched genes. Using a networking approach [33,34], we identified clusters of genes whose transcripts are significantly differentially transcribed (four-fold) between female and male adults of *H. contortus*. The totals of 459 female-specific and 2,354 male-specific genes represent 397 (degree: 10) and 1,620 (degree: 10) cluster hubs, respectively (Figure 2; see Additional file 1, Table S12). We found that both female and male gene sets were enriched for genes associated with growth, genital, embryonic, and germline development, and reproduction. Within the female set were genes associated with germline (for example, *cdc-25.2, glp-1, plk-2*, and *rpn-1*), oogenesis or egg laying (for example, *car-1, daf-4, epi-1, ima-3, mpk-1, ptp-2, rme-2*, and *sos-1*), embryogenesis (for example, *nhr-25, rab-7, unc-130, unc-6, spk-1, let-92, *and *let-767*), vulval development (for example, *let-60, lin-11, *and *rab-8*), and other reproductive and biological processes. Notable within the male set were genes associated specifically with spermatogenesis/sperm (for example, *alg-4 [tag-76], cyk-4, fer-1, hsp-12.2, hsp-12.3, spe-15, vab-1*, and *vpr-1*). There are at least 977 sex-enriched genes (34.7%) in *H. contortus *that do not have homologs in other organisms.

### Parasite-host interactions

Considering the substantial attack against *H. contortus *within the host, many ES proteins are expected to play crucial roles during parasite establishment, infection, immune modulation, or evasion. This expectation is supported by abundant transcription in the L4 and adult stages of genes encoding peptidases (*n *= 142), SCP-like extracellular proteins (including 20 neutrophil inhibitory factors; NIFs), lectins (*n *= 23), TTL proteins (*n *= 10), peptidase inhibitors (*n *= 6; including 4 Kunitz-like molecules) and fatty acid retinoid binding proteins (*n *= 4; see Additional file 1, Tables S10 and S13). In total, 333 of 1,457 genes (22.9%) encoding ES proteins were transcribed at significantly higher levels in the parasitic compared with the free-living stages (see Additional file 1, Table S10). The genome-wide average for this upregulation was significantly lower (14.7%; *P *= 5.8 × 10^-12^).

In the hematophagous stages, we identified 54 upregulated genes encoding SCP/TAPS proteins [35], characterized by one or more SCP-like extracellular domains (IPR014044 and/or IPR001283). These proteins, originally found in hookworms, are also called activation-associated proteins or *Ancylostoma*-secreted proteins (ASPs) [36,37]. Although the numbers of genes inferred to express SCP/TAPS proteins were similar between the L4 and adult stages (see Additional file 1, Tables S11 and S14), there were qualitative and quantitative differences in transcription compared with other developmental stages. Although two genes encoding the SCP/TAPS proteins Hc24 and Hc40 were identified previously in ES products of adult *H. contortus *[38,39], we identified 82 more such genes (see Additional file 1, Table S14). This finding supports a previous proposal for a wide array of molecules of this group in *H. contortus *[40], and suggests a diversified, active, and specific involvement of SCP/TAPS proteins in infection. Of the 84 SCP/TAPS proteins (62 single and 22 double SCP-like domain molecules) encoded by *H. contortus*, 74 were found to have homologs in *C. elegans *(see Additional file 1, Table S14); the 10 *H. contortus*-unique SCP/TAPS proteins, some of which are upregulated in parasitic stages, probably relate to host interactions and/or disease. Although SCP/TAPS proteins are still enigmatic, in terms of their functions, they deserve detailed investigation, particularly given that they are being explored as vaccine candidates for other hematophagous nematodes. For instance, in the human hookworm *Necator americanus, Na-*ASP-2 was tested in a phase I clinical trial, owing to its known protective properties in humans [41], although initial vaccination with the recombinant protein in adjuvant resulted in unexpected allergic responses following natural exposure to the parasite [42]. The crystal structure of *Na-*ASP-2 shows charge segregation similar to that of mammalian chemokines, indicating that this molecule might be an agonist or ligand for some GPCRs, such as chemokine receptors [43]. Of the 84 SCP/TAPS proteins identified in *H. contortus*, 20 were NIFs and predicted to be abundant in ES products, and have already been found in some other nematodes [19]. Although NIFs have not been reported previously for *H. contortus*, an *Ancylostoma caninum *homolog (SCP-1) is known to bind host integrin CR3 (CD11b/CD18) and to be able to inhibit neutrophil function, including oxidative burst [44,45].

As expected from previous molecular studies [40,46], eight genes encoding NIM-like proteins were found to be abundant in the hematophagous stages of *H. contortus *(see Additional file 1, Table S13). Although the functional roles of NIM proteins are unclear, they are likely to be involved in host-parasite interactions, because they are abundantly transcribed in parasitic stages. Most have N-terminal signal peptides and, thus appear to be actively excreted/secreted [40], although there is variation in the abundance of these proteins among different populations of *H. contortus *[40,46,47].

Of 53 genes encoding TTL proteins, 10 were significantly upregulated in parasitic stages of *H. contortus *(see Additional file 1, Table S13). Most TTL proteins identified to date are relatively conserved across large evolutionary distances [48], and are enzymes of purine catabolism that catalyze the conversion of 5-hydroxyisourate to 2-oxo-4-hydroxy-4-carboxy-5-ureidoimidazoline [49,50]. In metazoans, TTLs can also bind hormones, such as thyroxine (T4) and vitamin A [51], and can enable cell corpse engulfment by binding surface-exposed phosphatidylserine on apoptotic cells [52]. Among the proteins encoded by nematode-specific genes, TTLs represent one of the largest groups [53,54]. A subset of TTL proteins has also been identified in *Ostertagia ostertagi*, a nematode related to *H. contortus *[55,56], in the human filarial nematode *B. malayi *[57], and in the plant-parasitic nematodes *Xiphinema index, Heterodera glycines, Meloidogyne incognita*, and *Radopholus similis *[53,58-60]. For example, in *O. ostertagi*, at least 18 *ttl *genes have been identified by data mining, most of them being constitutively transcribed from the free-living L3 through to adult females and males [56]. In *H. contortus*, a TTL has been isolated from ES products from adult worms and shown to be immunogenic [40], and TTL homologs are also abundant in *An. caninum *ES [61]. These data suggest the testable hypothesis that TTLs, together with SCP/TAPS proteins, play key roles in host interactions.

### Immune responses

Based on the current knowledge and understanding of immune responses against helminths in animals [19], we compiled a comprehensive list of *H. contortus *ES homologs with known immunomodulatory or immunogenic roles in other nematodes (see Additional file 1: Table S13). Such homologs upregulated in the L4 and adult stages represent 5.6% of the predicted *H. contortus *secretome, which is significantly lower than the genome-wide average of 14.7% (*P*<10^-6^). In addition to the molecules HcES15 and HcES24, whose precise functions are still unclear, proteins within this secretome that are predicted to direct or suppress immune responses include close homologs of N-acteylglycosaminyltransferase and leucyl aminopeptidase ES-62 of the filarioid nematode *Acanthocheilonema vitae *[19]. ES-62 is known to inhibit B-cell, T-cell and mast cell proliferation/responses, induce a Th2 response through the inhibition of IL-12p70 production by dendritic cells, and promote alternative activation of the host macrophages via the inhibition of Toll-like receptor (TLR) signaling [19]. Other molecules of *H. contortus *predicted to be immunomodulatory include homologs of another B-cell inhibitor (CYS-1), 8 serpins and 20 NIFs [19]. Some *H. contortus *ES proteins are predicted to be involved in immune evasion; for instance, some could mask parasite antigens by mimicking host molecules (for example, C-type lectins, concanavalin A and galectins) [19]. In spite of some similarities among nematode-host systems, based on the nature and extent of molecules identified, the host immune responses against the parasitic stages of *H. contortus *appear to be distinct from those associated with other nematodes, such as *Ascaris *and filarioids, which is supported by other experimental findings [19]. Taken together, the present findings indicate that *H. contortus *has a substantial arsenal of ES proteins that are likely to be involved in modulating, evading, and/or blocking immune responses in the host.

### Vaccine molecules

There has been a major emphasis on the development of vaccines to fight against haemonchosis [6]. Most effort has been directed at inducing immunity in sheep against proteins expressed in or excreted/secreted from the gut of *H. contortus*, with the aim of disrupting or inhibiting the parasite's digestion of host blood. To date, the two most effective immunogens assessed have been the aminopeptidase family H11 [62,63] and the *Haemonchus *galactose-containing glycoprotein complex (H-gal-GP) [64]. Both of these molecular complexes contain integral membrane proteins with hemoglobinase activity, are expressed mainly in the microvillar surface of the parasite's gut, and induce 70 to 90% protection against infection in a number of sheep breeds [6]. In the current study, using genomic and transcriptomic data, we were able to define the different molecular variants within these two complexes.

We found that H11 represents a group of 25 different metallopeptidases (clan MA; family M01; see Additional file 1, Table S15), which are upregulated six-fold to 210-fold in the parasitic over the free-living stages of *H. contortus*. Key components of H-gal-GP, representing predominantly metallopeptidases (for example, MEPs 1 to 4) [65,66], aspartyl peptidases (for example, HcPEPs 1 and 2) [67,68], and cysteine peptidases (for example, AC-1 to AC-5; HMCP-1 to HMCP-6) [22,67,69-71], were also identified using sequence data from previous proteomic studies (see Additional file 1: Table S15). Again, as expected from previous studies [6], all three classes of peptidases were significantly upregulated in the L4 and adult stages (see Additional file 1, Table S15). We found substantial diversity in the cysteine peptidases (*n *= 81), which have been also under close scrutiny as vaccine candidates. Many of these enzymes (*n *= 14) represent clan C01A (cathepsin B-like peptidases), and 34.6% were represented in the ES degradome (see Additional file 1, Tables S4 and S16). We also identified 11 legumains (clan CD; family C13), which might activate key family C01A peptidases through cleavage of the peptide backbone between the pro-segment and mature enzyme domains [72].

In addition, the serine peptidase complex contortin has received attention as an efficient anticoagulant (with dipeptidyl IV activity) in parasitic stages of *H. contortus *[73]. Contortin is inferred to belong to clan SC serine peptidases (family S28). We found 13 family S28 representatives among the 107 serine peptidases predicted for *H. contortus *(see Additional file 1, Table S15), all of which were upregulated in the parasitic stages. Nine of these thirteen lysosomal Pro-Xaa carboxypeptidases were represented in the ES degradome (see Additional file 1, Table S16), supporting the contention that contortin is also immobilized [73]. Interestingly, *H. contortus *shares many of these key classes of peptidases with other hematophagous parasites, including hookworms, indicating relative conservation in sequence and function linked mainly to feeding (blood meal digestion or anticoagulation). Studies to date have shown that selected recombinant proteins representing H11 and H-gal-GP do not induce protective immune responses, and carbohydrate moieties alone are also not protective [6]. Therefore, the combined use of proteomic and glycomic tools, underpinned by the present genomic and transcriptomic data sets as well as by animal experimentation, should be advantageous for designing future vaccines.

### Prediction and prioritization of drug targets

The excessive and uncontrolled use of a small number of drug classes for the treatment of haemonchosis has led to major problems of drug resistance in *H. contortus *to most of these compounds [5]. Unfortunately, only a very small number of new anthelmintics (cyclooctodepsipeptides and aminoacetylnitriles) have been discovered in the past two decades using traditional chemical screening approaches [7,74]. Genome-guided drug target or drug discovery provides an alternative means to conventional screening and repurposing [75]. The aim of genome-guided discovery is to identify genes or molecules whose inactivation by one or more drugs will selectively kill parasites but not harm the host animal. Because *H. contortus *and related strongylid nematodes are challenging to maintain outside of their hosts, and gene-specific perturbation by double-stranded RNA interference (RNAi) is inconsistent [76], directly assessing gene essentiality on a large scale is not yet practical. However, essentiality can be predicted from functional information (for example, lethality) for *C. elegans*, and this approach has already yielded credible targets for nematocides [77]. For *H. contortus*, we inferred 641 molecules with essential homologs in *C. elegans *linked to lethal phenotypes upon gene silencing (see Additional file 1, Table S17). We also screened for enzymatic chokepoints in biological pathways of *H. contortus*. Such chokepoints represent reactions that consume or uniquely produce a molecular compound; the disruption of such enzymes should lead to a toxic accumulation (for unique substrates) or starvation (for unique products) of metabolites within cells [78,79]. We gave the highest priority to targets inferred to be encoded by single genes, reasoning that lower allelic variability in *H. contortus *populations would be less likely to give rise to drug resistance. Using this stringent approach, we predicted 260 druggable proteins in *H. contortus *(see Additional file 1, Table S17), of which 106 had ligands fulfilling the Lipinsky rule of five [80] (Table 3). Conspicuous among these were 17 channels or transporters, which represent protein classes known to be targets for anthelmintics, including macrocyclic lactones, levamisoles, and AADs [7,81,82], and other candidates including 27 kinases, 7 TFs, and 4 phosphatases known to be specific targets for norcantharidin analogues [77]. This list of prioritized target candidates could be tested for anti-nematodal effects in larval development assays or directly in experimental sheep, and should enable rational anthelmintic design.

**Table 3:** Druggable candidates encoded in the *Haemonchus contortus *draft

Group of proteins	Classification (number of molecules)	Total number
Kinases	CAMK (5), TKL (3), tyrosine protein kinases (3), AGC (2), CK1 (2), CMGC (2), STE (1), others (9)	27
Phosphatases	Fructose-1,6-bisphosphatase I (1), PP2A (1), PP2A-B (1), uridine phosphorylase (1)	4
GTPases	Rho (1)	1
Various enzymes	Replication and repair (9), hydrolases (5), lyases (5), transferases (5), oxidoreductases (3), translation (3), aminotransferase (2), cellular antigens (2), chaperones and folding catalysts (2), GTP-binding proteins (2), ligases (2), ubiquitin system (2), cyclins (1), cytoskeleton (1), fatty acid synthase (1), spliceosome (1), others (6)	53
		
Transporters and channels	Primary active transporters (10), incompletely characterized transport systems (3), electrochemical potential-driven transporters (2), group translocators (2)	17
Transcription factors	Helix-loop-helix (1), helix-turn-helix (4), zinc-coordinating DNA domain (2)	7
RNAi machinery	Proteins DCR-1 (2) and XPO-1 (1), all involved in small RNA biosynthesis	3
GPCRs	Class A (1)	1

### Prospects for functional genomics

**Figure 3:**
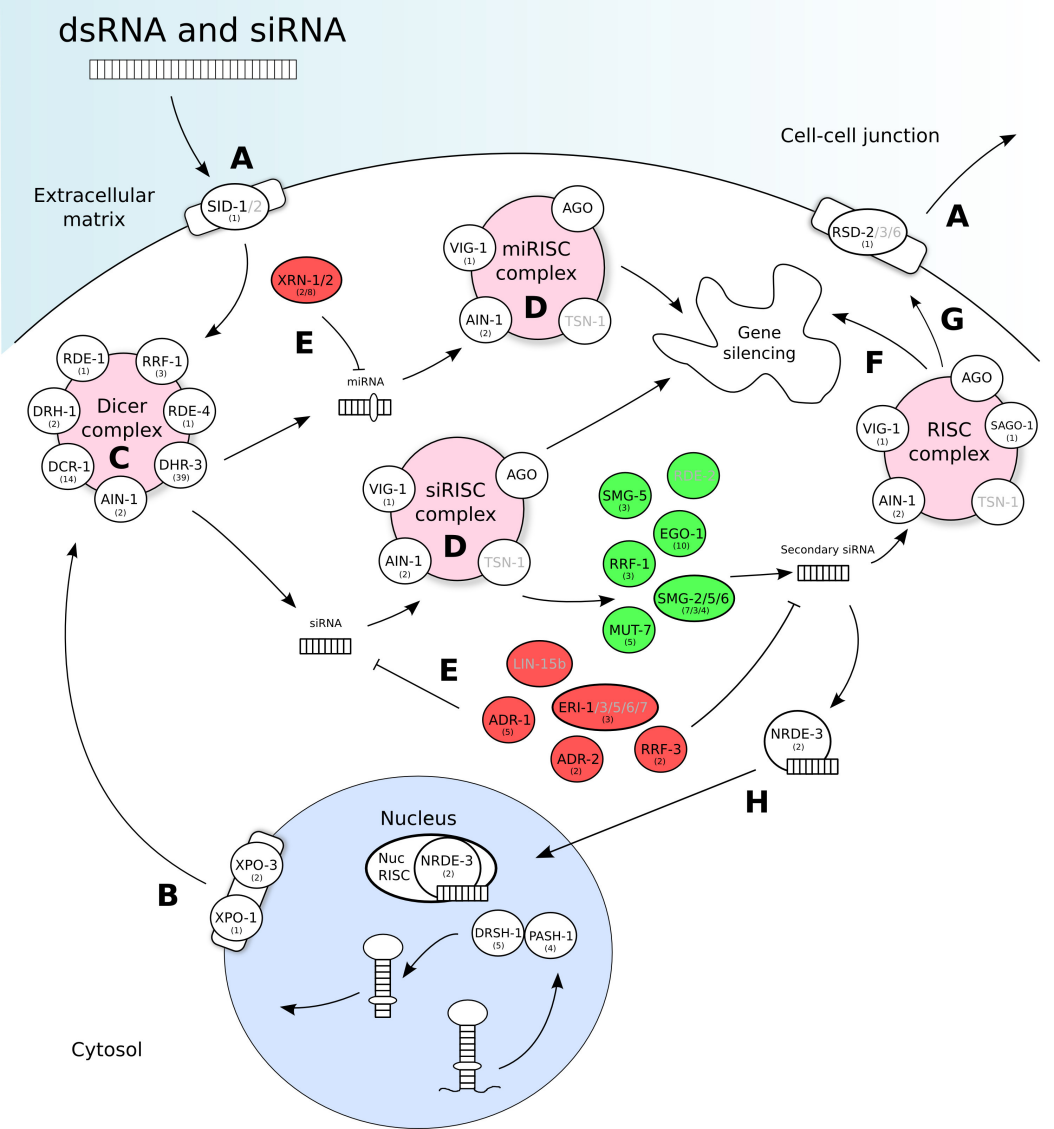
**Proposed RNA interference (RNAi) pathway of *Haemonchus contortus***. The genes predicted in *H. contortus *contain all of the previously identified core functional groups in nematode RNAi machinery [85], small RNA biosynthesis, double-stranded RNA (dsRNA) uptake and spreading, catalytic components, argonauts (AGO) of the RNA-induced silencing complex (RISC), RNAi inhibitors, and nuclear effectors. Genes present in *H. contortus *are represented by black codes, and those common to nematode RISC machinery are in gray. **(A) **Exogenous dsRNA and small interfering RNA (siRNA) enters cells via transporter SID-1. Internally produced secondary siRNA is spread to other cells via the transporter RSD-2. **(B) **Endogenous pre-microRNAs (pre-miRNAs) and siRNAs are produced in the nucleus, and exported to the cytosol via the nuclear export receptors XPO-1 and XPO-3. (**C**) Both the exogenous dsRNA and endogenous pre-miRNAs are cleaved by a dicer complex to produce siRNA and mature miRNA, respectively. **(D) **These RNAs are then bound to RISC, resulting in mRNA destruction or translational repression. **(E) **RNAi inhibitors can downregulate both siRNAs and miRNAs. **(F) **Secondary siRNAs produced by the catalytic components (MUT, SMF, and RRF) can contribute to downregulation of the target transcript. These siRNAs can also spread to other cells via **(G) **transporter RSD-2, and can be imported into the nucleus by **(H) **NRDE-3, in which they integrate to nuclear RISC to silence nascent RNA transcripts.

Genomic-guided drug discovery would be assisted by assessing essentiality of drug targets directly in *H. contortus *itself. Likewise, functional analysis of the approximately 30% of *H. contortus *genes that are parasite-specific, some of which are likely to play key roles in host-parasite interactions, would also be enabled by such gene inactivation. However, to date, gene-specific silencing in the parasite itself has been plagued by inconsistent results [76]. Recent findings suggest that this challenge can be overcome if the conditions for effective RNAi were optimized [83,84]. Inconsistent RNAi in *H. contortus *is apparently due to inefficient double-stranded RNA delivery, incomplete knowledge of the RNAi machinery, and variability in gene transcription in different stages or tissues of the parasite. Using the gene set of *H. contortus*, we identified 229 genes encoding proteins involved in the RNAi pathway (Figure 3; see Additional file 1, Table S18), including *rde-4 *and *rsd-2*, both previously thought to be absent [85], although we did not find *rde-2 *or *sid-2*. We also found that most RNAi genes in *H. contortus *are upregulated at the L2, L4, and adult stages (see Additional file 1, Table S18). These findings suggest that future assessments of gene function in *H. contortus *should focus on using these stages, which are most likely to be amenable to RNAi.

## Conclusions

The genomic and transcriptomic exploration of *H. contortus *provides new insights into the molecular biology of one of the most important parasites of small ruminants worldwide. This investigation has elucidated transcriptional alterations taking place throughout the life cycle, particularly during the transition from the free-living to the parasitic stages, and has emphasized molecules involved in host-parasite interactions and immune responses. Determining the genome sequence and transcriptomes of *H. contortus *can accelerate post-genomic explorations of genes and gene products involved in nematode development and reproduction, future proteomic and metabolomic studies, parasite-host interactions, and pathogenesis of disease. The characterization of the RNAi machinery for *H. contortus *also provides a solid platform for functional genomic work in selected stages of the parasite. Therefore, an integrated systems biology approach should provide novel strategies for parasite intervention via drugs, vaccines, and diagnostic tests. For instance, future work could focus on defining a spectrum of key molecules involved in pathways linked to the development of the nervous system in different stages of *H. contortus*, and assessing their potential as drug targets. Moreover, exploring unique groups of molecules, such as SCP/TAPS, TTLs, and the complex array of peptidases, and understanding the roles of these molecules in host-parasite interactions is likely to support the design of new interventions.

The complexity of the genome of *H. contortus *very probably relates to substantial sequence heterogeneity in non-coding regions among individual worms in the populations used for sequencing, and possibly even within individual worms. Although it is unclear why there is so much sequence variation in some genomic regions, it seems that this parasite mutates at a very high rate in non-coding regions, which might explain, then to some extent, the parasite's ability to rapidly become resistant to anthelmintics. To date, much research has focused on investigating possible associations between resistant phenotypes and mutations in particular candidate genes. Having available a draft genome of *H. contortus *now provides a solid foundation for genome-wide studies to identify genetic loci associated with anthelmintic resistance, and to explore their inheritance and mechanisms of drug resistance. Such studies will require an improved understanding of the population biology and genetics of this parasite, and knowledge of how mutations arise and are inherited. We expect drug resistance in *H. contortus *to be multigenic, and we hypothesize that complex resistance mechanisms operate in this nematode, possibly even involving microRNAs. Clearly, the genome of *H. contortus *will underpin future research in these and many other areas. Although the present study focused on *H. contortus*, the findings and the technological approaches used will be applicable to other parasitic nematodes of major animal and human health importance. Importantly, this first draft genome for a strongylid nematode paves the way for a rapid acceleration in our understanding of a wide range of socioeconomically important parasites of one of the largest nematode orders.

## Materials and methods

### Production and procurement of *H. contortus *

Animal ethics approval (no. 0707528) was granted by the University of Melbourne. *H. contortus *was produced in Merino lambs (3 months of age; Victoria, Australia) maintained under helminth-free conditions. Sheep were inoculated intraruminally (via oral intubation) with 5,000 to 10,000 infective L3s of *H. contortus*. Eggs were isolated from the feces of infected sheep (1 month after inoculation) using a sucrose flotation procedure [86]. L1s, L2s, and L3s were produced in culture (at 27°C), as described previously [87]. L1s, L2s, and L3s, identified according to Veglia [4], were collected after 1, 4 and 8 days, respectively, and washed extensively in tap water. L4s and adults of *H. contortus *were collected from the abomasa of infected lambs following euthanasia by intravenous injection of pentobarbitone sodium (Virbac, Carros Cedex, France) 13 days and 1 month, respectively, after infection with L3s. These latter two developmental stages of *H. contortus *were washed extensively in physiological saline, and males and females separated before freezing. All of the developmental stages of *H. contortus *collected were snap-frozen in liquid nitrogen and then stored at -70°C until use.

### RNA sequencing and transcriptome assembly

Total RNA was isolated separately from different developmental stages (egg, L1 to L4, and adult) and sexes (male and female) of *H. contortus *(Haecon-5 strain, Australia) using TriPure isolation reagent (Roche Molecular Biochemicals, Mannheim, Germany). For L1 to L3, packed volumes of 20 to 50 µl were used, equating to thousands of larvae. For L4 and adult stages, packed volumes of 50 to 200 µl were used, usually equating to 100 to 200 worms per aliquot. RNA yields were estimated spectrophotometrically (NanoDrop 1000; NanoDrop/Thermo Scientific Inc., Pittsburgh PA, USA), and the integrity of RNA was verified using a BioAnalyzer 2100 (Agilent Technologies Inc., Wilmington, DE, USA). RNA sequencing (RNA-seq) was carried out as described previously [88]. The sequences derived from each library representing each stage and sex were assessed for quality, and adaptors removed. Following removal of any potentially contaminating sequences, RNA-seq data for all stages and both sexes were assembled into *de novo *predicted transcripts using the programs Velvet and Oases [89] or SOAPdenovo [90]. Non-homologous transcripts were first used to train the *de novo *gene prediction programs SNAP [91] and AUGUSTUS [92], and transcripts were then used to assist the evidence-based prediction of the non-redundant gene set for *H. contortus*.

### Genomic sequencing and initial assembly

High molecular weight genomic DNA was isolated from adult male and female *H. contortus *(McMaster strain, Australia) using an established protocol [93]. The specificity of genomic DNA was verified by automated sequencing of the second internal transcribed spacer (ITS-2) of nuclear ribosomal DNA following PCR amplification from genomic DNA. Total DNA amounts were determined using a Qubit Fluorometer dsDNA HS Kit (Invitrogen), in accordance with the manufacturer's instructions. Genomic DNA integrity was verified by agarose gel electrophoresis and using a 2000 BioAnalyzer (Agilent). Mate-pair genomic libraries (with 300 bp and 500 bp inserts) were built [88], and checked for both size distribution and quality with a 2100 BioAnalyzer (Agilent). Jumping genomic libraries (with 2, 5, and 10 kb inserts; see Supplementary Table 1) were constructed as described previously [94]. To produce sufficient amounts of DNA for the jumping libraries, 250 to 500 ng of genomic DNA were subjected to whole genome amplification (WGA) using the REPLI-g Midi Kit (Qiagen Inc., Valencia, CA, USA), in accordance with the manufacturer's protocol. All sequencing was carried out on Illumina machines (either GA II or HiSeq; Illumina Inc., San Diego, CA, USA) with 2 × 75 or 2 × 100 reads for paired-end libraries, and 2 × 49 reads for jumping libraries. For all sequencing, reads were exported to FASTQ format [95]. Several steps were taken to enforce read quality. Custom Perl scripts were used to trim the final nucleotide of each read, nucleotides with a quality score of less than 3, or 'N' residues. Quality-trimmed reads were kept if they were 65 nt or more long from paired-end data or 48 nt or more long from jumping-library data.

We used a modified version of the read-decontamination pipeline of Kumar and Blaxter [96] to rid the genomic and RNA-seq datasets of any possible contaminating sequences of mammalian, bacterial, mycotic, protistan, and plant origins. In brief, genomic and RNA-seq reads were assembled into preliminary contigs using SOAPdenovo, without scaffolding for genomic DNA and using oases with scaffolding for RNA-seq. For genomic DNA contigs and cDNA scaffolds, minimum contig sizes of 60 and 200 nt, respectively, were accepted.

We mapped reads to the preliminary contigs with the program Bowtie 2 [97,98]. Unlike Kumar and Blaxter [96], we then performed exhaustive MegablastN [99] searches on all contigs (rather than using a random subset) to determine which sequences had likely contaminant status. MegablastN searching was done against opposing custom nematode and contaminant genomic DNA databases: the nematode set represented genomic assemblies from *C. elegans, P. pacificus, A. suum *(from WormBase WS230), and *Ancylostoma ceylanicum *(Schwarz EM, unpublished data). The contaminant set included sheep and cow genomic sequences, 1,991 bacterial genomes (including *Prevotella ruminicola*) from the European Nucleotide Archive (ENA), and a bovine rumen metagenome [100]. Because *A. ceylanicum *is a strongylid nematode parasite, related to *H. contortus *[101], we expected that any *H. contortus *contigs of genuine nematode origin were highly likely to have a better MegablastN hit to *A. ceylanicum *or *C. elegans *than to any contaminants. Each preliminary *H. contortus *contig was thus classed as a contaminant if it had a score against the contaminant database of 50 bits or more, and which was at least 50 bits higher than any match by that contig against the nematode database.

We exported all reads that failed to map to a contaminant contig (including both the reads that mapped to non-contaminant contigs and those that did not map to a contig at all). This set of reads was then used for genome and transcriptome assembly, and for quantifying transcription levels. Although our pipeline for decontamination is similar to that of Kumar and Blaxter [96] and uses much of the same source code, it differs by not trying to classify contigs as contaminants based on GC percentage or coverage levels (which we found not to work well with our data), but by using exhaustive MegablastN searching instead.

Our genomic reads, even after initial quality filtering, could not be assembled with Velvet because they required more than 256 GB of system RAM, the maximum amount available to us on our largest server. Therefore, for Velvet assembly, we used *khmer *to digitally normalize read frequencies [11]. First, we constructed a hash table of 75 GB in size, scanned through the paired-end genomic reads, and discarded reads with 20-mers that we had already found 50 times in previous reads. We rescanned the reads, discarding those with unique 20-mers, reasoning that unique 20-mers in such a large dataset were likely to represent sequencing errors or trace contaminants; *khmer *estimated the false positive rate of the hash table to be less than 0.001. The *khmer *filtering automatically converted the reads from FASTQ to FASTA format. We assembled *khmer*-filtered reads into a *H. contortus *genome sequence with Velvet 1.2 [102]. For our final Velvet assembly, velveth was run with k = 21; for preliminary assemblies, velveth was run with values from k = 41 down to k = 19. The velvetg parameters were as follows: -*shortMatePaired3 yes -shortMatePaired4 yes -shortMatePaired5 yes -cov_cutoff 4 -exp_cov 100 -min_contig_lgth 200 -ins_length 300 -ins_length_sd 50 -ins_length2 500 -ins_length2_sd 200 -ins_length3 2000 -ins_length4 5000 -ins_length5 10000*. Because Velvet 1.2 prioritizes paired-end over jumping-library data, artifactual long-range connections were less likely to confound assembly.

Preliminary assemblies with k-mer values at or near 41 were approximately 700 Mb in size, more than twice the estimate of the genome size (315 Mb) of *H. contortus *based on Feulgen image analysis densitometry [103]. The *khmer *filtering allowed us to complete assemblies with k values as low as 21. With decontaminated reads, k = 21 resulted in an assembly size of 404 Mb, perhaps because using unusually small word values allowed Velvet's de Bruin graph to merge polymorphisms rather than treat them as distinct, allelic sequences; scaffold N50 was 13.6 kb. The k = 21 assembly showed relatively low levels of non-scaffolding residues (79.3% non-N), but we improved this percentage to 95.8% non-N residues by adding reads to the assembly with GapCloser from SOAPdenovo. GapCloser also modestly increased the assembly size to 414 Mb and scaffold N50 to 17.6 kb.

To improve the Velvet assembly, we used SOAPdenovo (version 2.0) to scaffold the 404 Mb assembly using error-corrected reads without *khmer *filtering. The program GapCloser was used to close gaps in the scaffolded assembly. With k = 21, this gave us an assembly of size 453 Mb that achieved an N50 of 34.2 kb after gap-closure, with 93.8% non-N residues. For both Velvet and SOAPdenovo, we tested the gap-filled k = 21 assemblies using the program CEGMA [104]. For Velvet, we predicted 157 of 248 conserved eukaryotic genes (CEGs; 63%) completely, and 211 of 248 (85%) at least partially. Using SOAPdenovo, we predicted 182 of 248 CEGs (73%) completely, and 232 of 248 (91.5%) at least partially. Given the superior N50, completeness of assembly and the prediction of more CEGs, we selected the SOAPdenovo scaffolded assembly.

### Final draft genome assembly

The initial assembly (453 Mb) was substantially larger than the genome size estimate based on Feulgen image analysis densitometry (315 Mb). Therefore, we re-evaluated the genomic sequence composition by comparing assembled DNA scaffolds containing at least one predicted protein-coding gene against assembled DNA scaffolds that had no such prediction. For this comparison, we did not use only the approximately 26,000 protein-coding genes in our final set, which all had RNA-seq evidence to support their expression, but instead used a larger set of approximately 29,184 genes, which included both RNA-seq-supported and protein-supported predictions. Moreover, we mapped Illumina sequencing reads that had been decontaminated but not yet subjected to digital normalization with *khmer*, so that variations in sequencing coverage would remain detectable. We found that scaffolds containing protein-coding genes had a much higher coverage of Illumina sequencing reads (with a single distinct peak) than scaffolds that were completely devoid of predicted protein-coding genes (which were represented by a very low-coverage peak). All the high-coverage coding regions were contained within a total of 320 Mb of scaffolded sequences, whereas all of the low-coverage non-coding regions were within a total of 133 Mb of scaffolded sequences. Moreover, when we examined the two sequence sets with CEGMA for completeness of gene content, the 320 Mb set was essentially identical to the 453 Mb assembly, whereas the 133 Mb set was almost completely devoid of gene content. We therefore selected the 320 Mb scaffold set as our final draft assembly. Low-coverage scaffolds might represent a residue from the *khmer *removal of sequences with high coverage, and heterozygosity/heterogeneity/haplotype differences linked to non-coding regions, possibly due to variations among individual worms of the population.

### Identification and annotation of non-coding regions and protein-coding genes

Genomic repeats specific to *H. contortus *were modeled using the program RepeatModeler [105] by merging repeat predictions by RECON [106] and RepeatScout [107]. Repeats in the *H. contortus *genome assembly were identified by RepeatMasker [108] using modeled repeats (via RepeatModeler) and known repeats in Repbase (version 17.02) [109].

The *H. contortus *protein-coding gene set was inferred using an integrative approach, utilizing the transcriptomic data for all stages and both sexes sequenced in the present study. First, all 185,706 contigs representing the combined transcriptome for *H. contortus *were run through BLAT [110] and filtered for full-length open reading frames (ORFs), ensuring the validity of splice sites. These ORFs were then used to train the *de novo *gene prediction programs SNAP [91] and AUGUSTUS [92] by producing a hidden Markov model (HMM) for each program. The same ORFs were also given (as an expressed sequence tag (EST) input) to MAKER2 [111] to provide evidence for predicted genes. In addition, all raw reads representing the combined *H. contortus *transcriptome were run through the programs TopHat and Cufflinks to provide additional information on transcripts and on exon-intron boundaries in the form of a Generic Feature Format (GFF) file. HMMs, the EST input, and the GFF file were subjected to analysis using MAKER2 to provide a consensus set of 27,782 genes for *H. contortus*. Genes inferred to encode peptides of 30 or more amino acids in length were preserved, resulting in the prediction of a total of 27,135 genes. To account for the genes in DNA repeat regions, identified by RepeatMasker, we removed genes (*n *= 1,028) that overlapped these regions by at least one nucleotide and did not have a similarity match (BLASTp; e-value ≤10^-5^) [112] with genes of *C. elegans*. Following filtering of the predicted genes by Annotation Edit Distance (AED <0.4) [113], the final set was inferred to contain 23,610 protein-coding genes. The predicted genes were represented by amino acid and cDNA sequences.

### Functional annotation of all predicted protein sequences

First, following prediction of the protein-coding gene set for *H. contortus*, each inferred amino acid sequence was assessed for conserved protein domains using InterProScan [114,115], employing default settings. Second, amino acid sequences were subjected to BLASTp (e-value ≤10^-5^) against the following protein databases: *C. elegans *in WormBase [116]; Swiss-Prot and TrEMBL within UniProtKB [117]; Kinase SARfari [118] and the protein kinase database for *C. elegans *[119], which contains all domain information for *C. elegans *kinases [120]; GPCR SARfari [118]; Transporter Classification Database [121,122]; KEGG [123,124]; LGICs [125]; ChEMBL [126]; NCBI protein nr [127]; and an in-house RNAi machinery database for nematodes. Finally, the BLASTp results were used to infer key protein groups, including peptidases, kinases, phosphatases, GTPases, GPCRs, channel and transporter proteins, TFs, major sperm proteins, vitellogenins, SCP/TAPS proteins, and RNAi machinery proteins.

Each coding gene was assessed against the known KEGG Orthology (KO) term BLAST hits. These BLAST hits were clustered to a known protein family using the KEGG-BRITE hierarchy in a custom script. ES proteins were predicted using SignalP (version 4.0) [128] and TMHMM (version 2.0c) [122,129,130] and by BLASTp homology searching of the validated Signal Peptide Database [131] and of an ES database containing published proteomic data for *A. suum *[14], *B. malayi *[15]. *C. elegans *[116], and *T. spiralis *[16]. In the final annotation, proteins inferred from genes were classified based on a homology match (e-value cut-off, ≤10^-5^) to: (i) a curated, specialist protein database, followed by (ii) the KEGG database, followed by (iii) the Swiss-Prot database, followed by (iv) the annotated gene set for a model organism, including *C. elegans*, followed by (v) a recognized, conserved protein domain based on InterProScan analysis. Any inferred proteins lacking a match (e-value cut-off, ≤10^-5^) in at least one of these analyses were designated hypothetical proteins. The final annotated protein-coding gene set for *H. contortus *is available for download at WormBase [116] in nucleotide and amino acid formats.

### Differential transcription analysis

The analysis of empirical RNA-seq data for the developmental stages and sexes of *H. contortus *was conducted using edgeR [132], an R programming language [133] package. Trimmomatic software [134], using the parameters *phred64, ILLUMINACLIP:illuminaClipping.fa:2:40:20*,*LEADING:3, TRAILING:3, SLIDINGWINDOW:4:20, MINLEN:40*, was used to filter the paired-end RNA-seq reads for quality in individual samples (representing egg, L1, L2, L3, and L4 males, L4 females, and adult males and adult females).

Each set of the decontaminated and quality-filtered paired-end RNA-seq data was mapped to the set of cDNAs using Burrows-Wheeler Aligner software [135]. The numbers of mapped reads per individual gene were extracted using the program SAMtools [136]. The resultant read counts per developmental stage were used as input data for edgeR. Initially, the levels of differential transcription data were calculated by pairwise comparison of stages in the life cycle of *H. contortus *(for example, egg versus L1; L1 versus L2; L2 versus L3; L3 versus L4; L3 versus adult; L4 versus adult) and of sexes (L4 female versus L4 male; adult female versus adult male). Using edgeR dispersion factor zero, the genes were considered differentially transcribed if the logarithmic change in fold change (FC) compared with the normalized read count data was greater than or equal to 2 and the *P*-value was less than or equal to 10^-5^. The levels of differential transcription data were then calculated by pairwise comparisons between all free-living (egg, L1, L2. and L3) and parasitic (L4 and adult) stages. The genes were considered differentially transcribed, using edgeR-calculated common and gene-wise dispersion factors, if the FC compared with the normalized read count data was greater than or equal to 2 and the false discovery rate (FDR) was less than or equal to 0.05. To identify abundant sex-enriched genes in adult *H. contortus*, more stringent criteria (FC ≥ 4; FDR ≤ 0.05) were applied. The resultant differentially transcribed genes were subjected to genetic interaction network analysis [33,34], based on the pre-calculated, weighed interactions among *C. elegans *genes. Hubs with at least 10 interactions (degree ≥ 10) among different genes were considered significant.

### Protein homology

Homologs between *H. contortus *and *A. suum, B. malayi, C. elegans*, and *T. spiralis *were inferred by comparison of all proteins by BLASTp (e-value ≤ 10^-5^), pairing proteins based on reciprocal best hits, and inferring homologous groups from pairs using a custom script.

### Prediction of essentiality, chokepoints, and drug targets

Essentiality was inferred by filtering *C. elegans *homologs (BLASTp; e-value ≤ 10^-5^) [112] representing lethal phenotype in RNAi experiments listed in WormBase release WS222 [116]. The metabolic chokepoints were predicted from essential genes with a unique match to the combined identifier of KEGG pathway and KO group. KEGG pathways and KO groups were inferred from the KEGG database (BLASTp; e-value ≤10^-5^). The molecules in metabolic chokepoints that satisfied Lipinski's rule of five in ChEMBL were identified from matches with target molecules (BLASTp; e-value ≤10^-30^) in the ChEMBL database.

### Additional bioinformatic and data analyses, and use of software for document preparation

Data analysis was conducted in a Unix environment or Microsoft Excel 2007 using standard commands. Bioinformatic scripts required to facilitate data analysis were designed using mainly the Python 2.6 scripting language.

### Data availability

The genomic sequence and gene predictions for *H. contortus *are available in WormBase. The genome sequence has also been deposited at DDBJ/EMBL/GenBank (accession number AUUS00000000), and genomic and RNA-seq reads in the NCBI short read archive (SRA; accession numbers SRP027504 and SRP026668, respectively).

## Abbreviations

AAD: aminoacetonitrile derivative; AED: Annotation Edit Distance; ASP: *Ancylostoma*-secreted protein; CEGMA: Core Eukaryotic Genes Mapping Approach; ES: Excretory/secretory; EST: expressed sequence tag; FC: fold change; FDR: false discovery rate; GFF: Generic Feature Format; GPCR: G protein-coupled receptor; H-gal-GP: *Haemonchus *galactose-containing glycoprotein complex; HMM: hidden Markov model; KEGG: Kyoto Encyclopedia of Genes and Genomes; KO: KEGG Orthology; L1 to L4: First-stage to fourth-stage larvae; LGIC: Ligand-gated ion channel; LINE: long interspersed nuclear element; LTR: long terminal repeat; NIF: Neutrophil inhibitory factor; ORF: open reading frame; RTE: retrotransposable element; RNAi: RNA interference; SCP/TAPS: Sperm-coating protein/Tpx-1/Ag5/PR-1/Sc7; SINE: short interspersed nuclear element; TF: Transcription factor; Th: T helper; TTL: transthyretin-like; VIC: voltage-gated channel.

## Authors' contributions

RBG, BEC, and AJ produced *H. contortus *in sheep and isolated genomic DNA; BEC and NDY isolated RNA; BAW constructed RNA-seq libraries; IA optimized Illumina protocols for paired-end libraries. CTB, ACH, and JP devised *khmer *filtering; CTB and EMS ran it on quality-filtered genomic reads. EMS decontaminated all reads *in silico*. EMS and PKK performed genomic assembly and annotation; PKK, BEC, NDY, ARJ, and RSH performed transcriptomic analyses. TRG estimated the genome size of *H. contortus *by Feulgen image analysis densitometry. RBG, EMS, PKK, and PWS wrote the manuscript, with crucial contributions from NDY, ARJ, and inputs from XQZ, PRB, and other co-authors. RBG and PWS managed the project. All authors read and approved the final manuscript.

## Supplementary Material

Additional file 1 **Supplementary tables S1-S18**. Click here for file

## References

[B1] Bethony JM Loukas A Hotez PJ Knox DP Vaccines against blood-feeding nematodes of humans and livestock. Parasitology 2006 14 Suppl S63 79 17274849 10.1017/S0031182006001818

[B2] Nikolaou S Gasser RB Prospects for exploring molecular developmental processes in *Haemonchus contortus*. Int J Parasitol 2006 14 859 868 16759659 10.1016/j.ijpara.2006.04.007

[B3] Scott I Sutherland I Gastrointestinal Nematodes of Sheep and Cattle: Biology and Control 2010 Oxford: Wiley-Blackwell

[B4] Veglia F The anatomy and life-history of the *Haemonchus contortus *(Rud.). Rep Dir Vet Res 1915 14 347 500

[B5] Kaplan RM Vidyashankar AN An inconvenient global truth worming and anthelmintic resistance. Vet Parasitol 2012 14 70 78 22154968 10.1016/j.vetpar.2011.11.048

[B6] Knox D Proteases in blood-feeding nematodes and their potential as vaccine candidates. Adv Exp Med Biol 2011 14 155 176 21660664 10.1007/978-1-4419-8414-2_10

[B7] Kaminsky R Ducray P Jung M Clover R Rufener L Bouvier J Weber SS Wenger A Wieland-Berghausen S Goebel T Gauvry N Pautrat F Skripsky T Froelich O Komoin-Oka C Westlund B Sluder A Mäser P A new class of anthelmintics effective against drug-resistant nematodes. Nature 2008 14 176 180 18337814 10.1038/nature06722

[B8] von Samson-Himmelstjerna G Harder A Sangster NC Coles GC Efficacy of two cyclooctadepsipeptides, PF1022A and emodepside, against anthelmintic-resistant nematodes in sheep and cattle. Parasitology 2005 14 343 347 15796017 10.1017/s0031182004006523

[B9] Little PR Hodge A Maeder SJ Wirtherle NC Nicholas DR Cox GG Conder GA Efficacy of a combined oral formulation of derquantel-abamectin against the adult and larval stages of nematodes in sheep, including anthelmintic-resistant strains. Vet Parasitol 2011 14 180 193 21684691 10.1016/j.vetpar.2011.05.008

[B10] Pell J Hintze A Canino-Koning R Howe A Tiedje JM Brown CT Scaling metagenome sequence assembly with probabilistic de Bruijn graphs. Proc Natl Acad Sci USA 2012 14 13272 13277 22847406 10.1073/pnas.1121464109 PMC3421212

[B11] Brown CT Howe A Zhang Q Pyrkosz AB Brom TH A reference-free algorithm for computational normalization of shotgun sequencing data. NASA ADS 2012 eprint arXiv:1203.4802 [q-bio.GN]

[B12] C elegans Sequencing Consortium C Genome sequence of the nematode *C. elegans *a platform for investigating biology. Science 1998 14 2012 2018 9851916 10.1126/science.282.5396.2012

[B13] Dieterich C Clifton SW Schuster LN Chinwalla A Delehaunty K Dinkelacker I Fulton L Fulton R Godfrey J Minx P Mitreva M Roeseler W Tian H Witte H Yang SP Wilson RK Sommer RJ The *Pristionchus pacificus *genome provides a unique perspective on nematode lifestyle and parasitism. Nat Genet 2008 14 1193 1198 18806794 10.1038/ng.227 PMC3816844

[B14] Jex AR Liu S Li B Young ND Hall RS Li Y Yang L Zeng N Xu X Xiong Z Chen F Wu X Zhang G Fang X Kang Y Anderson GA Harris TW Campbell BE Vlaminck J Wang T Cantacessi C Schwarz EM Ranganathan S Geldhof P Nejsum P Sternberg PW Yang H Wang J Wang J Gasser RB *Ascaris suum *draft genome. Nature 2011 14 529 533 22031327 10.1038/nature10553

[B15] Ghedin E Wang S Spiro D Caler E Zhao Q Crabtree J Allen JE Delcher AL Guiliano DB Miranda-Saavedra D Angiuoli SV Creasy T Amedeo P Haas B El-Sayed NM Wortman JR Feldblyum T Tallon L Schatz M Shumway M Koo H Salzberg SL Schobel S Pertea M Pop M White O Barton GJ Carlow CK Crawford MJ Daub J Draft genome of the filarial nematode parasite *Brugia malayi*. Science 2007 14 1756 1760 17885136 10.1126/science.1145406 PMC2613796

[B16] Mitreva M Jasmer DP Zarlenga DS Wang Z Abubucker S Martin J Taylor CM Yin Y Fulton L Minx P Yang SP Warren WC Fulton RS Bhonagiri V Zhang X Hallsworth-Pepin K Clifton SW McCarter JP Appleton J Mardis ER Wilson RK The draft genome of the parasitic nematode *Trichinella spiralis*. Nat Genet 2011 14 228 235 21336279 10.1038/ng.769 PMC3057868

[B17] Spanier B Sturzenbaum SR Holden-Dye LM Baumeister R *Caenorhabditis elegans *neprilysin NEP-1: an effector of locomotion and pharyngeal pumping. J Mol Biol 2005 14 429 437 16081104 10.1016/j.jmb.2005.06.063

[B18] McKerrow JH Caffrey C Kelly B Loke P Sajid M Proteases in parasitic diseases. Annu Rev Pathol 2006 14 497 536 18039124 10.1146/annurev.pathol.1.110304.100151

[B19] Hewitson JP Grainger JR Maizels RM Helminth immunoregulation: the role of parasite secreted proteins in modulating host immunity. Mol Biochem Parasitol 2009 14 1 11 19406170 10.1016/j.molbiopara.2009.04.008 PMC2706953

[B20] Baugh LR Demodena J Sternberg PW RNA Pol II accumulates at promoters of growth genes during developmental arrest. Science 2009 14 92 94 19251593 10.1126/science.1169628

[B21] Cox GN Shamansky LM Boisvenue RJ *Haemonchus contortus*: evidence that the 3A3 collagen gene is a member of an evolutionarily conserved family of nematode cuticle collagens. Exp Parasitol 1990 14 175 185 2404780 10.1016/0014-4894(90)90098-w

[B22] Pratt D Cox GN Milhausen MJ Boisvenue RJ A developmentally regulated cysteine protease gene family in *Haemonchus contortus*. Mol Biochem Parasitol 1990 14 181 191 2090940 10.1016/0166-6851(90)90143-a

[B23] Williamson AL Lecchi P Turk BE Choe Y Hotez PJ McKerrow JH Cantley LC Sajid M Craik CS Loukas A A multi-enzyme cascade of hemoglobin proteolysis in the intestine of blood-feeding hookworms. J Biol Chem 2004 14 35950 35957 15199048 10.1074/jbc.M405842200

[B24] Ranjit N Zhan B Stenzel DJ Mulvenna J Fujiwara R Hotez PJ Loukas A A family of cathepsin B cysteine proteases expressed in the gut of the human hookworm, *Necator americanus*. Mol Biochem Parasitol 2008 14 90 99 18501979 10.1016/j.molbiopara.2008.04.008

[B25] Ranjit N Zhan B Hamilton B Stenzel D Lowther J Pearson M Gorman J Hotez P Loukas A Proteolytic degradation of hemoglobin in the intestine of the human hookworm *Necator americanus*. J Infect Dis 2009 14 904 912 19434933 10.1086/597048

[B26] Roos MH Tielens AG Differential expression of two succinate dehydrogenase subunit-B genes and a transition in energy metabolism during the development of the parasitic nematode *Haemonchus contortus*. Mol Biochem Parasitol 1994 14 273 281 7808477 10.1016/0166-6851(94)90154-6

[B27] Skuce PJ Stewart EM Smith WD Knox DP Cloning and characterization of glutamate dehydrogenase (GDH) from the gut of *Haemonchus contortus*. Parasitology 1999 14 297 304 10205806 10.1017/s0031182098003850

[B28] Fetterer RH Hill DE Rhoads ML Characterization of a hemoglobin-like protein from adult *Haemonchus contortus*. J Parasitol 1999 14 295 300 10219312

[B29] Blaxter ML Nemoglobins divergent nematode globins. Parasitol Today 1993 14 353 360 15463668 10.1016/0169-4758(93)90082-q

[B30] Liddell S Knox DP Extracellular and cytoplasmic Cu/Zn superoxide dismutases from *Haemonchus contortus*. Parasitology 1998 14 Pt 4 383 394 9585940 10.1017/s0031182098002418

[B31] Kotze AC Catalase induction protects *Haemonchus contortus *against hydrogen peroxide in vitro. Int J Parasitol 2003 14 393 400 12705932 10.1016/s0020-7519(03)00012-2

[B32] van Rossum AJ Jefferies JR Rijsewijk FA LaCourse EJ Teesdale-Spittle P Barrett J Tait A Brophy PM Binding of hematin by a new class of glutathione transferase from the blood-feeding parasitic nematode *Haemonchus contortus*. Infect Immun 2004 14 2780 2790 15102788 10.1128/IAI.72.5.2780-2790.2004 PMC387910

[B33] Zhong W Sternberg PW Genome-wide prediction of *C. elegans *genetic interactions. Science 2006 14 1481 1484 16527984 10.1126/science.1123287

[B34] Lee I Lehner B Crombie C Wong W Fraser AG Marcotte EM A single gene network accurately predicts phenotypic effects of gene perturbation in *Caenorhabditis elegans*. Nat Genet 2008 14 181 188 18223650 10.1038/ng.2007.70 PMC13030915

[B35] Cantacessi C Campbell BE Visser A Geldhof P Nolan MJ Nisbet AJ Matthews JB Loukas A Hofmann A Otranto D Sternberg PW Gasser RB A portrait of the "SCP/TAPS" proteins of eukaryotes--developing a framework for fundamental research and biotechnological outcomes. Biotechnol Adv 2009 14 376 388 19239923 10.1016/j.biotechadv.2009.02.005

[B36] Hawdon JM Jones BF Hoffman DR Hotez PJ Cloning and characterization of Ancylostoma-secreted protein. A novel protein associated with the transition to parasitism by infective hookworm larvae. J Biol Chem 1996 14 6672 6678 8636085 10.1074/jbc.271.12.6672

[B37] Datu BJ Loukas A Cantacessi C O'Donoghue P Gasser RB Investigation of the regulation of transcriptional changes in *Ancylostoma caninum *larvae following serum activation, with a focus on the insulin-like signalling pathway. Vet Parasitol 2009 14 139 148 19054616 10.1016/j.vetpar.2008.10.026

[B38] Schallig HD van Leeuwen MA Cornelissen AW Protective immunity induced by vaccination with two *Haemonchus contortus *excretory secretory proteins in sheep. Parasite Immunol 1997 14 447 453 9372512 10.1046/j.1365-3024.1997.d01-148.x

[B39] Rehman A Jasmer DP A tissue specific approach for analysis of membrane and secreted protein antigens from *Haemonchus contortus *gut and its application to diverse nematode species. Mol Biochem Parasitol 1998 14 55 68 9879887 10.1016/s0166-6851(98)00132-7

[B40] Yatsuda AP Krijgsveld J Cornelissen AW Heck AJ de Vries E Comprehensive analysis of the secreted proteins of the parasite *Haemonchus contortus *reveals extensive sequence variation and differential immune recognition. J Biol Chem 2003 14 16941 16951 12576473 10.1074/jbc.M212453200

[B41] Bethony JM Simon G Diemert DJ Parenti D Desrosiers A Schuck S Fujiwara R Santiago H Hotez PJ Randomized, placebo-controlled, double-blind trial of the *Na*-ASP-2 hookworm vaccine in unexposed adults. Vaccine 2008 14 2408 2417 18396361 10.1016/j.vaccine.2008.02.049

[B42] Diemert DJ Pinto AG Freire J Jariwala A Santiago H Hamilton RG Periago MV Loukas A Tribolet L Mulvenna J Correa-Oliveira R Hotez PJ Bethony JM Generalized urticaria induced by the *Na*-ASP-2 hookworm vaccine: implications for the development of vaccines against helminths. J Allergy Clin Immunol 2012 14 169 176 e166 22633322 10.1016/j.jaci.2012.04.027

[B43] Asojo OA Goud G Dhar K Loukas A Zhan B Deumic V Liu S Borgstahl GE Hotez PJ X-ray structure of *Na*-ASP-2, a pathogenesis-related-1 protein from the nematode parasite, *Necator americanus*, and a vaccine antigen for human hookworm infection. J Mol Biol 2005 14 801 814 15713464 10.1016/j.jmb.2004.12.023

[B44] Moyle M Foster DL McGrath DE A hookworm glycoprotein that inhibits neutrophil function is a ligand of the integrin CD11b/CD18. J Biol Chem 1994 14 10008 10015 7908286

[B45] Rieu P Sugimori T Griffith DL Arnaout MA Solvent-accessible residues on the metal ion-dependent adhesion site face of integrin CR3 mediate its binding to the neutrophil inhibitory factor. J Biol Chem 1996 14 15858 15861 8663417 10.1074/jbc.271.27.15858

[B46] Geldhof P Whitton C Gregory WF Blaxter M Knox DP Characterisation of the two most abundant genes in the *Haemonchus contortus *expressed sequence tag dataset. Int J Parasitol 2005 14 513 522 15826643 10.1016/j.ijpara.2005.02.009

[B47] Skuce PJ Yaga R Lainson FA Knox DP An evaluation of serial analysis of gene expression (SAGE) in the parasitic nematode, *Haemonchus contortus*. Parasitology 2005 14 553 559 15991498 10.1017/s0031182004006973

[B48] Rahat O Yitzhaky A Schreiber G Cluster conservation as a novel tool for studying protein-protein interactions evolution. Proteins 2008 14 621 630 17972288 10.1002/prot.21749

[B49] Lee Y Lee DH Kho CW Lee AY Jang M Cho S Lee CH Lee JS Myung PK Park BC Park SG Transthyretin-related proteins function to facilitate the hydrolysis of 5-hydroxyisourate, the end product of the uricase reaction. FEBS Lett 2005 14 4769 4774 16098976 10.1016/j.febslet.2005.07.056

[B50] Ramazzina I Folli C Secchi A Berni R Percudani R Completing the uric acid degradation pathway through phylogenetic comparison of whole genomes. Nat Chem Biol 2006 14 144 148 16462750 10.1038/nchembio768

[B51] Li X Buxbaum JN Transthyretin and the brain re-visited is neuronal synthesis of transthyretin protective in Alzheimer's disease? Mol Neurodegener 2011 14 79 22112803 10.1186/1750-1326-6-79 PMC3267701

[B52] Wang X Li W Zhao D Liu B Shi Y Chen B Yang H Guo P Geng X Shang Z Peden E Kage-Nakadai E Mitani S Xue D *Caenorhabditis elegans *transthyretin-like protein TTR-52 mediates recognition of apoptotic cells by the CED-1 phagocyte receptor. Nat Cell Biol 2010 14 655 664 20526330 10.1038/ncb2068 PMC2896453

[B53] Jacob J Vanholme B Haegeman A Gheysen G Four transthyretin-like genes of the migratory plant-parasitic nematode *Radopholus similis *members of an extensive nematode-specific family. Gene 2007 14 9 19 17765408 10.1016/j.gene.2007.07.015

[B54] Parkinson J Mitreva M Whitton C Thomson M Daub J Martin J Schmid R Hall N Barrell B Waterston RH McCarter JP Blaxter ML A transcriptomic analysis of the phylum Nematoda. Nat Genet 2004 14 1259 1267 15543149 10.1038/ng1472

[B55] Vercauteren I Geldhof P Peelaers I Claerebout E Berx G Vercruysse J Identification of excretory-secretory products of larval and adult *Ostertagia ostertagi *by immunoscreening of cDNA libraries. Mol Biochem Parasitol 2003 14 201 208 12615319 10.1016/s0166-6851(02)00274-8

[B56] Saverwyns H Visser A Van Durme J Power D Morgado I Kennedy MW Knox DP Schymkowitz J Rousseau F Gevaert K Vercruysse J Claerbout E Geldhof P Analysis of the transthyretin-like (TTL) gene family in *Ostertagia ostertagi*--comparison with other strongylid nematodes and *Caenorhabditis elegans*. Int J Parasitol 2008 14 1545 1556 18571174 10.1016/j.ijpara.2008.04.004

[B57] Hewitson JP Harcus YM Curwen RS Dowle AA Atmadja AK Ashton PD Wilson A Maizels RM The secretome of the filarial parasite, *Brugia malayi*: proteomic profile of adult excretory-secretory products. Mol Biochem Parasitol 2008 14 8 21 18439691 10.1016/j.molbiopara.2008.02.007

[B58] Gao B Allen R Maier T Davis EL Baum TJ Hussey RS The parasitome of the phytonematode *Heterodera glycines*. MPMI 2003 14 720 726 12906116 10.1094/MPMI.2003.16.8.720

[B59] McCarter JP Mitreva MD Martin J Dante M Wylie T al. e Analysis and functional classification of transcripts from the nematode *Meloidogyne incognita*. Genome Biol 2004 14 R26 10.1186/gb-2003-4-4-r26 PMC154577 12702207

[B60] Furlanetto C Cardle L Brown DJF Jones JT Analysis of expressed sequence tags from the ectoparasitic nematode *Xiphinema index*. Nematology 2005 14 95 104

[B61] Mulvenna J Hamilton B Nagaraj SH Smyth D Loukas A Gorman JJ Proteomics analysis of the excretory/secretory component of the blood-feeding stage of the hookworm, *Ancylostoma caninum*. Mol Cell Proteomics 2009 14 109 121 18753127 10.1074/mcp.M800206-MCP200

[B62] Munn EA A helical, polymeric extracellular protein associated with the luminal surface of *Haemonchus contortus *intestinal cells. Tissue Cell 1977 14 23 34 898175 10.1016/0040-8166(77)90046-5

[B63] Newton SE Munn EA The development of vaccines against gastrointestinal nematode parasites, particularly *Haemonchus contortus*. Parasitol Today 1999 14 116 122 10322325 10.1016/s0169-4758(99)01399-x

[B64] Smith SK Smith WD Immunisation of sheep with an integral membrane glycoprotein complex of *Haemonchus contortus *and with its major polypeptide components. Res Vet Sci 1996 14 1 6 8745246 10.1016/s0034-5288(96)90121-6

[B65] Redmond DL Knox DP Newlands G Smith WD Molecular cloning and characterisation of a developmentally regulated putative metallopeptidase present in a host protective extract of *Haemonchus contortus*. Mol Biochem Parasitol 1997 14 77 87 9108550 10.1016/s0166-6851(96)02812-5

[B66] Newlands GF Skuce PJ Nisbet AJ Redmond DL Smith SK Pettit D Smith WD Molecular characterization of a family of metalloendopeptidases from the intestinal brush border of *Haemonchus contortus*. Parasitology 2006 14 357 368 16740178 10.1017/S0031182006000217

[B67] Longbottom D Redmond DL Russell M Liddell S Smith WD Knox DP Molecular cloning and characterisation of a putative aspartyl proteinase associated with a gut membrane protein complex from adult *Haemonchus contortus*. Mol Biochem Parasitol 1997 14 63 72 9274868 10.1016/s0166-6851(97)00074-1

[B68] Smith WD Skuce PJ Newlands GFJ Smith SK Pettit D Aspartyl proteases from the intestinal brush border of *Haemonchus contortus *as protective antigens for sheep. Parasite Immunol 2003 14 521 530 15053773 10.1111/j.0141-9838.2004.00667.x

[B69] Cox GN Pratt D Hageman R Boisvenue RJ Molecular cloning and primary sequence of a cysteine protease expressed by *Haemonchus contortus *adult worms. Mol Biochem Parasitol 1990 14 25 34 2385265 10.1016/0166-6851(90)90093-2

[B70] Pratt D Armes LG Hageman R Reynolds V Boisvenue RJ Cox GN Cloning and sequence comparisons of four distinct cysteine proteases expressed by *Haemonchus contortus *adult worms. Mol Biochem Parasitol 1992 14 209 218 1574079 10.1016/0166-6851(92)90071-q

[B71] Skuce PJ Redmond DL Liddell S Stewart EM Newlands GF Smith WD Knox DP Molecular cloning and characterization of gut-derived cysteine proteinases associated with a host protective extract from *Haemonchus contortus*. Parasitology 1999 14 405 412 10581619 10.1017/s0031182099004813

[B72] Dalton JP Brindley PJ Donnelly S Robinson MW The enigmatic asparaginyl endopeptidase of helminth parasites. Trends Parasitol 2009 14 59 61 19101207 10.1016/j.pt.2008.11.002

[B73] Geldhof P Knox D The intestinal contortin structure in *Haemonchus contortus*: an immobilised anticoagulant? Int J Parasitol 2008 14 1579 1588 18599060 10.1016/j.ijpara.2008.05.002

[B74] Harder A Schmitt-Wrede HP Krücken J Marinovski P Wunderlich F Willson J Amliwala K Holden-Dye L Walker R Cyclooctadepsipeptides - an anthelmintically active class of compounds exhibiting a novel mode of action. Int J Antimicrob Ag 2003 14 318 331 10.1016/s0924-8579(03)00219-x 13678839

[B75] Shanmugam D Ralph SA Carmona SJ Crowther GJ Roos DS Agüero F Integrating and Mining Helminth Genomes to Discover and Prioritize Novel Therapeutic Targets 2012 Wiley Online Library Wiley, USA

[B76] Geldhof P Visser A Clark D Saunders G Britton C Gilleard J Berriman M Knox D RNA interference in parasitic helminths: current situation, potential pitfalls and future prospects. Parasitology 2007 14 609 619 17201997 10.1017/S0031182006002071

[B77] Campbell BE Tarleton M Gordon CP Sakoff JA Gilbert J McCluskey A Gasser RB Norcantharidin analogues with nematocidal activity in *Haemonchus contortus*. Bioorg Med Chem Lett 2011 14 3277 3281 21536433 10.1016/j.bmcl.2011.04.031

[B78] Yeh I Hanekamp T Tsoka S Karp PD Altman RB Computational analysis of *Plasmodium falciparum *metabolism: organizing genomic information to facilitate drug discovery. Genome Res 2004 14 917 924 15078855 10.1101/gr.2050304 PMC479120

[B79] Berriman M Haas BJ LoVerde PT Wilson RA Dillon GP Cerqueira GC Mashiyama ST Al-Lazikani B Andrade LF Ashton PD Aslett MA Bartholomeu DC Blandin G Caffrey CR Coghlan A Coulson R Day TA Delcher A DeMarco R Djikeng A Eyre T Gamble JA Ghedin E Gu Y Hertz-Fowler C Hirai H Hirai Y Houston R Ivens A Johnston DA The genome of the blood fluke *Schistosoma mansoni*. Nature 2009 14 352 U365 19606141 10.1038/nature08160 PMC2756445

[B80] Lipinski CA Lead- and drug-like compounds: the rule-of-five revolution. Drug Discov Today: Technol 2004 14 337 341 10.1016/j.ddtec.2004.11.007 24981612

[B81] Campbell WC Fisher MH Stapley EO Albers-Schonberg G Jacob TA Ivermectin: a potent new antiparasitic agent. Science 1983 14 823 828 6308762 10.1126/science.6308762

[B82] Qian H Robertson AP Powell-Coffman JA Martin RJ Levamisole resistance resolved at the single-channel level in *Caenorhabditis elegans*. FASEB J 2008 14 3247 3254 18519804 10.1096/fj.08-110502 PMC2518249

[B83] Samarasinghe B Knox DP Britton C Factors affecting susceptibility to RNA interference in *Haemonchus contortus *and *in vivo *silencing of an H11 aminopeptidase gene. Int J Parasitol 2011 14 51 59 20699100 10.1016/j.ijpara.2010.07.005

[B84] Selkirk ME Huang SC Knox DP Britton C The development of RNA interference (RNAi) in gastrointestinal nematodes. Parasitology 2012 14 605 612 22459433 10.1017/S0031182011002332

[B85] Dalzell JJ McVeigh P Warnock ND Mitreva M Bird DM Abad P Fleming CC Day TA Mousley A Marks NJ Maule AG RNAi effector diversity in nematodes. PLoS Negl Trop Dis 2011 14 e1176 21666793 10.1371/journal.pntd.0001176 PMC3110158

[B86] Mes TH Eysker M Ploeger HW A simple, robust and semi-automated parasite egg isolation protocol. Nat Protoc 2007 14 486 489 17406611 10.1038/nprot.2007.56

[B87] Nikolaou S Hartman D Presidente PJ Newton SE Gasser RB HcSTK, a *Caenorhabditis elegans *PAR-1 homologue from the parasitic nematode, *Haemonchus contortus*. Int J Parasitol 2002 14 749 758 12062493 10.1016/s0020-7519(02)00008-5

[B88] Mortazavi A Schwarz EM Williams B Schaeffer L Antoshechkin I Wold BJ Sternberg PW Scaffolding a *Caenorhabditis nematode *genome with RNA-seq. Genome Res 2010 14 1740 1747 20980554 10.1101/gr.111021.110 PMC2990000

[B89] Schulz MH Zerbino DR Vingron M Birney E Oases: robust de novo RNA-seq assembly across the dynamic range of expression levels. Bioinformatics 2012 14 1086 1092 22368243 10.1093/bioinformatics/bts094 PMC3324515

[B90] Li R Zhu H Ruan J Qian W Fang X Shi Z Li Y Li S Shan G Kristiansen K Li S Yang H Wang J Wang J *De novo *assembly of human genomes with massively parallel short read sequencing. Genome Res 2010 14 265 272 20019144 10.1101/gr.097261.109 PMC2813482

[B91] Korf I Gene finding in novel genomes. BMC Bioinformatics 2004 14 59 15144565 10.1186/1471-2105-5-59 PMC421630

[B92] Stanke M Tzvetkova A Morgenstern B AUGUSTUS at EGASP: using EST, protein and genomic alignments for improved gene prediction in the human genome. Genome Biol 2006 14 10.1186/gb-2006-7-s1-s11 PMC1810548 16925833

[B93] Gasser RB Hu M Chilton NB Campbell BE Jex AJ Otranto D Cafarchia C Beveridge I Zhu X Single-strand conformation polymorphism (SSCP) for the analysis of genetic variation. Nat Protoc 2006 14 3121 3128 17406575 10.1038/nprot.2006.485

[B94] Li R Fan W Tian G Zhu H He L Cai J Huang Q Cai Q Li B Bai Y Zhang Z Zhang Y Wang W Li J Wei F Li H Jian M Li J Zhang Z Nielsen R Li D Gu W Yang Z Xuan Z Ryder OA Leung FC Zhou Y Cao J Sun X Fu Y The sequence and *de novo *assembly of the giant panda genome. Nature 2010 14 311 317 20010809 10.1038/nature08696 PMC3951497

[B95] Cock PJ Fields CJ Goto N Heuer ML Rice PM The Sanger FASTQ file format for sequences with quality scores, and the Solexa/Illumina FASTQ variants. Nucleic Acids Res 2010 14 1767 1771 20015970 10.1093/nar/gkp1137 PMC2847217

[B96] Kumar S Blaxter ML Simultaneous genome sequencing of symbionts and their hosts. Symbiosis 2011 14 119 126 22448083 10.1007/s13199-012-0154-6 PMC3294205

[B97] Langmead B Trapnell C Pop M Salzberg SL Ultrafast and memory-efficient alignment of short DNA sequences to the human genome. Genome Biol 2009 14 R25 19261174 10.1186/gb-2009-10-3-r25 PMC2690996

[B98] Langmead B Salzberg SL Fast gapped-read alignment with Bowtie 2. Nat Methods 2012 14 357 359 22388286 10.1038/nmeth.1923 PMC3322381

[B99] Zhang Z Schwartz S Wagner L Miller W A greedy algorithm for aligning DNA sequences. J Comput Biol 2000 14 203 214 10890397 10.1089/10665270050081478

[B100] Hess M Sczyrba A Egan R Kim TW Chokhawala H Schroth G Luo S Clark DS Chen F Zhang T Mackie RI Pennacchio LA Tringe SG Visel A Woyke T Wang Z Rubin EM Metagenomic discovery of biomass-degrading genes and genomes from cow rumen. Science 2011 14 463 467 21273488 10.1126/science.1200387

[B101] Chilton NB Huby-Chilton F Gasser RB Beveridge I The evolutionary origins of nematodes within the order Strongylida are related to predilection sites within hosts. Mol Phylogenet Evol 2006 14 118 128 16584893 10.1016/j.ympev.2006.01.003

[B102] Zerbino DR Birney E Velvet: Algorithms for *de novo *short read assembly using de Bruijn graphs. Genome Res 2008 14 821 829 18349386 10.1101/gr.074492.107 PMC2336801

[B103] Hardie DC Gregory TR Hebert PD From pixels to picograms: a beginners' guide to genome quantification by Feulgen image analysis densitometry. J Histochem Cytochem 2002 14 735 749 12019291 10.1177/002215540205000601

[B104] Parra G Bradnam K Ning Z Keane T Korf I Assessing the gene space in draft genomes. Nucleic Acids Res 2009 14 289 297 19042974 10.1093/nar/gkn916 PMC2615622

[B105] Smit AFA Robert H Kas A Siegel A Gish W Price A Pevzner P RepeatModeler. 2011 1.0.5 Institute of Systems Biology

[B106] Bao Z Eddy SR Automated *de novo *identification of repeat sequence families in sequenced genomes. Genome Res 2002 14 1269 1276 12176934 10.1101/gr.88502 PMC186642

[B107] Price AL Jones NC Pevzner PA De novo identification of repeat families in large genomes. Bioinformatics 2005 14 Suppl 1 i351 358 15961478 10.1093/bioinformatics/bti1018

[B108] RepeatMasker Open-3.0 http://www.repeatmasker.org

[B109] Jurka J Kapitonov VV Pavlicek A Klonowski P Kohany O Walichiewicz J Repbase Update, a database of eukaryotic repetitive elements. Cytogenet Genome Res 2005 14 462 467 16093699 10.1159/000084979

[B110] Kent WJ BLAT - The BLAST-like alignment tool. Genome Res 2002 14 656 664 11932250 10.1101/gr.229202 PMC187518

[B111] Holt C Yandell M MAKER2: an annotation pipeline and genome-database management tool for second-generation genome projects. BMC Bioinformatics 2011 14 491 22192575 10.1186/1471-2105-12-491 PMC3280279

[B112] Altschul SF Gish W Miller W Myers EW Lipman DJ Basic local alignment search tool. J Mol Biol 1990 14 403 410 2231712 10.1016/S0022-2836(05)80360-2

[B113] Eilbeck K Moore B Holt C Yandell M Quantitative measures for the management and comparison of annotated genomes. BMC Bioinformatics 2009 14 67 19236712 10.1186/1471-2105-10-67 PMC2653490

[B114] Zdobnov EM Apweiler R InterProScan - an integration platform for the signature-recognition methods in InterPro. Bioinformatics 2001 14 847 848 11590104 10.1093/bioinformatics/17.9.847

[B115] Quevillon E Silventoinen V Pillai S Harte N Mulder N Apweiler R Lopez R InterProScan: protein domains identifier. Nucleic Acids Res 2005 14 W116 120 15980438 10.1093/nar/gki442 PMC1160203

[B116] Harris TW Antoschechkin I Bieri T Blasair D Chan J Chen WJ De La Cruz N Davis P Duesbury M Fang R Fernandes J Han M Kishore R Lee R Müller HM Nakamura C Ozersky P Percherski A Rangarajan A Rogers A Schindelman G Schwarz EM Tuli MA Van Auken K Wang D Wang X Williams G Yook K Durbin R Stein LD WormBase: a comprehensive resource for nematode research. Nucleic Acids Res 2010 14 D463 467 19910365 10.1093/nar/gkp952 PMC2808986

[B117] Magrane M the UniProt Consortium UniProt Knowledgebase: a hub of integrated protein data. Database (Oxford) 2011 14 bar009 21447597 10.1093/database/bar009 PMC3070428

[B118] Gaulton A Bellis LJ Bento AP Chambers J Davies M Hersey A Light Y McGlinchey S Michalovich D Al-Lazikani B Overington JP ChEMBL: a large-scale bioactivity database for drug discovery. Nucleic Acids Res 2012 14 D1100 1107 21948594 10.1093/nar/gkr777 PMC3245175

[B119] Plowman GD Sudarsanam S Bingham J Whyte D Hunter T The protein kinases of Caenorhabditis elegans: a model for signal transduction in multicellular organisms. Proc Natl Acad Sci USA 1999 14 13603 13610 10570119 10.1073/pnas.96.24.13603 PMC24111

[B120] Manning G Genomic overview of protein kinases. WormBook 2005 1 19 18050405 10.1895/wormbook.1.60.1 PMC4780929

[B121] Saier MH Jr Yen MR Noto K Tamang DG Elkan C The Transporter Classification Database: recent advances. Nucleic Acids Res 2009 14 D274 278 19022853 10.1093/nar/gkn862 PMC2686586

[B122] Saier MH Jr Tran CV Barabote RD TCDB: the Transporter Classification Database for membrane transport protein analyses and information. Nucleic Acids Res 2006 14 D181 186 16381841 10.1093/nar/gkj001 PMC1334385

[B123] Kanehisa M Goto S KEGG: kyoto encyclopedia of genes and genomes. Nucleic Acids Res 2000 14 27 30 10592173 10.1093/nar/28.1.27 PMC102409

[B124] Kanehisa M Goto S Sato Y Furumichi M Tanabe M KEGG for integration and interpretation of large-scale molecular datasets. Nucleic Acids Res 2012 14 D109 D114 22080510 10.1093/nar/gkr988 PMC3245020

[B125] Donizelli M Djite MA Le Novere N LGICdb: a manually curated sequence database after the genomes. Nucleic Acids Res 2006 14 D267 269 16381861 10.1093/nar/gkj104 PMC1347466

[B126] Bellis LJ Akhtar R Al-Lazikani B Atkinson F Bento AP Chambers J Davies M Gaulton A Hersey A Ikeda K Krüger FA Light Y McGlinchey S Santos R Stauch B Overington JP Collation and data-mining of literature bioactivity data for drug discovery. Biochem Soc Trans 2011 14 1365 1370 21936816 10.1042/BST0391365

[B127] NCBI Resource Coordinators N Database resources of the National Center for Biotechnology Information. Nucleic Acids Res 2013 14 D8 D20 23193264 10.1093/nar/gks1189 PMC3531099

[B128] Petersen TN Brunak S von Heijne G Nielsen H SignalP 4.0: discriminating signal peptides from transmembrane regions. Nat Methods 2011 14 785 786 21959131 10.1038/nmeth.1701

[B129] Sonnhammer EL von Heijne G Krogh A A hidden Markov model for predicting transmembrane helices in protein sequences. Proc Int Conf Intell Syst Mol Biol 1998 14 175 182 9783223

[B130] Krogh A Larsson B von Heijne G Sonnhammer EL Predicting transmembrane protein topology with a hidden Markov model: application to complete genomes. J Mol Biol 2001 14 567 580 11152613 10.1006/jmbi.2000.4315

[B131] Chen YJ Zhang Y Yin YB Gao G Li SG Jiang Y Gu XC Luo JC SPD - a web-based secreted protein database. Nucleic Acids Res 2005 14 D169 D173 15608170 10.1093/nar/gki093 PMC540047

[B132] Robinson MD McCarthy DJ Smyth GK edgeR: a Bioconductor package for differential expression analysis of digital gene expression data. Bioinformatics 2010 14 1 139 140 19910308 10.1093/bioinformatics/btp616 PMC2796818

[B133] R Development CoreTeam R R: A language and environment for statistical computing. R Foundation for Statistical Computing 2011 2.15 Vienna, Austria

[B134] Lohse M Bolger AM Nagel A Fernie AR Lunn JE Stitt M Usadel B RobiNA: a user-friendly, integrated software solution for RNA-Seq-based transcriptomics. Nucleic Acids Res 2012 14 W622 627 22684630 10.1093/nar/gks540 PMC3394330

[B135] Li H Durbin R Fast and accurate long-read alignment with Burrows-Wheeler transform. Bioinformatics 2010 14 589 595 20080505 10.1093/bioinformatics/btp698 PMC2828108

[B136] Li H Handsaker B Wysoker A Fennell T Ruan J Homer N Marth G Abecasis G Durbin R The Sequence Alignment/Map format and SAMtools. Bioinformatics 2009 14 2078 2079 19505943 10.1093/bioinformatics/btp352 PMC2723002

